# A potential mechanism for the sexual dimorphism in the onset of puberty and incidence of idiopathic central precocious puberty in children: sex-specific kisspeptin as an integrator of puberty signals

**DOI:** 10.3389/fendo.2012.00149

**Published:** 2012-12-13

**Authors:** Suzy D. C. Bianco

**Affiliations:** ^1^Department of Molecular and Cellular Pharmacology, University of Miami Miller School of MedicineMiami, FL, USA; ^2^Division of Endocrinology and Metabolism, Department of Medicine, University of Miami Miller School of MedicineMiami, FL, USA

**Keywords:** central precocious puberty, kisspeptin receptor signaling, reproduction, LH surge, sexual differentiation of the brain

## Abstract

The major determinants of the variability in pubertal maturation are reported to be genetic and inherited. Nonetheless, nutritional status contributes significantly to this variability. Malnutrition delays puberty whereas obesity has been associated to a rise in Idiopathic Central Precocious Puberty (ICPP) in girls. However, epidemiology data indicate that contribution of obesity to early puberty varies significantly among ethnic groups, and that obesity-independent inheritable genetic factors are the strongest predictors of early puberty in any ethnic group. In fact, two human mutations with confirmed association to ICPP have been identified in children with no history of obesity. These mutations are in kisspeptin and kisspeptin receptor, a ligand/receptor pair with a major role on the onset of puberty and female cyclicity after puberty. Progressive increases in kisspeptin expression in hypothalamic nuclei known to regulate reproductive function has been associated to the onset of puberty, and hypothalamic expression of kisspeptin is reported to be sexually dimorphic in many species, which include humans. The hypothalamus of females is programmed to express significantly higher levels of kisspeptin than their male counterparts. Interestingly, incidence of ICPP and delayed puberty in children is markedly sexually dimorphic, such that ICPP is at least 10-fold more frequent in females, whereas prevalence of delayed puberty is about 5-fold higher in males. These observations are consistent with a possible involvement of sexually dimorphic kisspeptin signaling in the sexual dimorphism of normal puberty and of pubertal disorders in children of all ethnicities. This review discusses the likelihood of such associations, as well as a potential role of kisspeptin as the converging target of environmental, metabolic, and hormonal signals, which would be integrated in order to optimize reproductive function.

## Introduction

A mystery that still puzzles scientists is what initiates puberty. The age at onset and duration of pubertal development is primarily driven by genetics (Palmert and Boepple, 2001; Palmert and Hirschhorn, 2003; Parent et al., 2003). However, timing of sexual maturation is influenced by other factors such as nutrition, environment, and sex (Parent et al., 2003). This review focuses on the role of the G protein-coupled kisspeptin receptor—KISS1R/Kiss1r—and its endogenous ligand, kisspeptin, on the onset of puberty and etiology of pubertal disorders. Potential roles of nutrition and sex on kisspeptin signaling are also discussed.

The onset of puberty is first detected as an increase in pulses and frequency of gonadotropin-releasing hormone (GnRH), which leads to mirroring increases in the secretion of gonadotropins luteinizing hormone (LH) and follicle-stimulating hormone (FSH) by the pituitary gland. Failure to increase GnRH or gonadotropin secretion at puberty is the underlying cause of idiopathic hypogonadotropic hypogonadism (IHH), which is characterized by impaired sexual maturation and infertility (Seminara et al., 1998). Conversely, premature activation of GnRH secretion leads to idiopathic gonadotropin-dependent (or central) precocious puberty (ICPP).

Most of the advances into the mechanisms of re-activation of the hypothalamic-pituitary-gonadal (HPG) or reproductive axis at puberty have been provided by the characterization of genetic mutations associated with reproductive disorders in humans. The majority of mutations studied to date were identified in patients with IHH, a disorder far less frequent than ICPP. Prevalence of premature puberty has been predicted to be 0.2% in one population (Teilmann et al., 2005), whereas incidence of IHH is estimated to be 1–10 cases per 100,000 births (or 0.001–0.01%) (Seminara et al., 1998). Despite of the higher prevalence and of an estimated 30% familial cases (de Vries et al., 2004), the first genetic mutation associated to ICPP was published just four years ago, in 2008 (Teles et al., 2008).

Two striking distinctions between ICPP and IHH are the inheritance mode and the marked sexually dimorphic distribution of these disorders. The pedigree of families with history of IHH (with a normal sense of smell) show or suggest an autosomal *recessive* mode of inheritance (Bianco and Kaiser, 2009). This was confirmed in reported cases, which show that only individuals carrying the associated mutation in the *homozygous* (or compound heterozygous) state exhibit the IHH phenotype, whereas *heterozygous* parents and siblings have no obvious reproductive abnormalities (de Roux et al., 2003; Seminara et al., 2003; Bhagavath et al., 2006; Topaloglu et al., 2006; Bedecarrats and Kaiser, 2007; Nimri et al., 2011). On the other hand, the pedigree of families with a history of ICPP indicate an autosomal *dominant* mode of transmission (de Vries et al., 2004) that is supported by two reports of human mutations with confirmed association with ICPP, which were identified in the *heterozygous* state in affected children (Teles et al., 2008; Silveira et al., 2010). Interestingly, females are at least ten times more likely to develop premature puberty than males (de Vries et al., 2004). This likelihood increases if only idiopathic cases of early puberty are considered (Teilmann et al., 2005; Teles et al., 2011). Conversely, incidence of hypogonadism is predicted to be about 5-fold elevated in males (Sykiotis et al., 2010). Severity of the hypogonadism is also influenced by sex as males quite often exhibit more severe symptoms than females carrying the same mutation.

## Sexual maturation of the brain

Sexual maturation of the brain is driven by exposure to sex steroids at specific windows of sensitivity during development. The stage of brain development at the time of exposure appears to be a critical determinant of brain *masculinization* or *feminization*. However, there is species specificity in the timing of brain sensitivity to these effects. In rodents, brain masculinization occurs just before and after birth and is heavily dependent on local conversion of androgens to estradiol by brain aromatases (McCarthy, 2008; McCarthy et al., 2009). In contrast, sexual maturation of the brain in humans and other primates occurs earlier in development, around mid- to late-gestation. Androgens (rather than estrogens) are the determinants of brain masculinization in primates, although brain aromatization also plays a role (Michael et al., 1987; Wallen, 2005). Manipulation of brain exposure to androgens during certain critical developmental windows may produce subjects with the genitals of one sex but with behaviors that are typical of the opposite sex (Wallen, 2005). Accordingly, abnormal prenatal exposure of female embryos to androgens has been described to result in male-typical behavior and decreased female-typical behavior in species such as Rhesus monkeys (Wallen, 2005), guinea pig (Phoenix et al., 1959) and rats (Simerly, 2002). Interestingly, abnormal exposure of mouse embryos to sex steroids has been associated to corresponding abnormalities in the sexual dimorphism of kisspeptin expression in the hypothalamus (Kauffman et al., 2007a,b; Gonzalez-Martinez et al., 2008).

Consequences of abnormal exposure of the developing human brain to sex steroids are evidenced by anomalous puberty, fertility, sexual behavior and even sexual identity in patients carrying certain genetic mutations (Deladoey et al., 1999; Cohen-Bendahan et al., 2005; Lin et al., 2007; Zirilli et al., 2008). A classical example is the brain masculinization that is consistently reported for girls born with congenital adrenal hyperplasia. Hyperplastic adrenal glands in affected girls produce excessive amounts of androgens, which flood the developing brains of affected females (Cohen-Bendahan et al., 2005). Additionally, affected girls can develop precocious puberty, an effect also observed in female rodents exposed to androgen (Witham et al., 2012). Precocious Puberty may sound inconsistent with masculinization or incomplete feminization of the female brain, which would rather be expected to cause delayed puberty. Nonetheless, premature puberty is a common symptom in girls with a family history of another disorder strongly associated to female androgenization: polycystic ovary syndrome (PCOS) (Franceschi et al., 2010). Elevated androgens in these cases could be contributing to premature puberty by abnormally stimulating the hypothalamic-pituitary-gonadal (HPG) or reproductive axis. Incidentally, serum kisspeptin is reported to be elevated in adolescents with PCOS. Also, serum kisspeptin positively correlated to serum LH and testosterone in affected adolescents, suggesting an involvement of kisspeptin in the etiology of PCOS in these girls (Chen et al., 2010b).

Genetic mutations leading to aromatase deficiency are also reported to cause abnormal sexual maturation of the brain in humans and in mice (Lin et al., 2007; Bakker et al., 2010). This is due to insufficient aromatization of androgens to estrogens resulting in elevated serum androgens. Among the cases of congenital aromatase deficiency in humans is the 46XX (female) who exhibited boy-typical behavior and male gender identity from early age, despite estrogen supplementation started at age 3 to correct for low bone density and delayed bone age (Lin et al., 2007). Similar masculinization of the brain is described for female mice with congenital aromatase insufficiency (Bakker et al., 2010). Interestingly, deficiencies of sexual differentiation of the brain in these mice were associated to an absence of sexual dimorphism in the hypothalamic expression of kisspeptin (Bakker et al., 2010).

## Sexual dimorphism of puberty and of pubertal disorders

During pubertal transition to sexual maturity, the reproductive axis is activated in humans (Delemarre-van de Waal, 2002) and other non-human primates (Plant et al., 1989). The pubertal rise in gonadal sex steroids is coordinated with the appearance of secondary sexual features, which culminates with the attainment of reproductive competence. The onset of puberty in girls occurs 1–2 years earlier than in boys; menarche happens even earlier than sexual maturity in boys (Iuliano-Burns et al., 2009). A similar phenomenum is observed in Rhesus monkeys: menarche occurs around age 2 years in females, whereas males only reach sexual maturity during the breeding season of their fourth year of age (Wilen and Naftolin, 1976; Resko et al., 1982; Mann et al., 1998). These observations are consistent with sexual dimorphism of the major drivers of puberty.

Prevalence of pubertal disorders in humans is also sexually dimorphic: incidence of precocious puberty is disproportionally higher in girls when compared to boys (de Vries et al., 2004). The ratio for idiopathic CPP is estimated to be 15–20 females for each male with the disorder (Teles et al., 2011). On the other hand, incidence of IHH is 5-fold elevated in males when compared to females (Seminara et al., 1998; Sykiotis et al., 2010).

The basis for the sexually dimorphic presentation of puberty and pubertal disorders in humans is not known, but the underlying mechanisms could involve sexually dimorphic signaling pathways with a role on GnRH secretion, rather than sexual dimporhism of GnRH neurons. This would be compatible with the involvement of KISS1R signaling, which has been shown to be sexually dimorphic in the hypothalamus of mice (Wray and Gainer, 1987; Kauffman et al., 2007b), rats (Kauffman et al., 2007a), and sheep (Schwanzel-Fukuda et al., 1981). On the other hand, GnRH neurons were not found to be sexually dimorphic in experimental animals such as rats or guinea pig (Clarkson and Herbison, 2006; Cheng et al., 2010). While there are no reports of sexually dimorphic pubertal disorders on experimental animals, investigation of animal models may help to elucidate the underlying causes of the dimorphism in humans, provided that appropriate consideration is given to species diversity.

Of note, requirement of GnRH neurons for fertility in mice has been reported to be sexually dimorphic (Herbison et al., 2008). The majority of GnRH neurons were not essential for puberty or fertility in male or female mice under ideal conditions (Herbison et al., 2008). Male mice expressing 12% of total GnRH neurons had normal reproductive function and fertility. Females with 12% of total GnRH neurons had normal puberty, but subsequently develop infertility due to an inability to generate LH surges and ovulate (Herbison et al., 2008). This demonstrated that females require additional GnRH neurons to be fertile after puberty. Sure enough, females expressing 34% of the GnRH neurons had no fertility or cyclicity problems later on (Herbison et al., 2008). The excess of GnRH neurons not required for puberty or fertility in ideal conditions could be, instead, required to warrant or modulate GnRH pulsatility in response to environmental, nutrition, stress, lactation or other cues conveying adverse situations. If this were the case, adaptive capability of GnRH responsiveness to adversity would be incredibly high, as the majority of GnRH fibers would not be required in ideal conditions.

## Idiopathic central precocious puberty (ICPP) facts

Gonadotropic-dependent or central precocious puberty (CPP) is characterized by early activation of the reproductive axis. This form of precocious puberty is further classified as *idiopathic* (ICPP) after tumors or other anatomical abnormalities are discarded (Klein, 1999). Thus, ICPP is early puberty of central origin with no obvious underlying cause. Dysfunction in these cases presumably lyes on signaling pathways regulating GnRH pulsatility, which is unmasked at puberty. Thus, ICPP cases are particularly interesting to investigate, as uncovering the underlying deffect may expose yet unkown or underappreciated brain pathways critical for puberty and/or reproductive competence.

Onset of puberty occurs earlier in girls, who are considerably more likley to develop precocious puberty, and 95% of girls reported with precocious puberty develop the idiopathic (or genetic) central form of the disorder (Klein, 1999). This sexual dimorphism could be associated to sexually dimorphic signals to puberty. The high rate of familial cases of ICPP emphasizes the genetic origin of this disorder (Palmert and Boepple, 2001; Anderson et al., 2003; Palmert and Hirschhorn, 2003; Parent et al., 2003; Prete et al., 2008; Aksglaede et al., 2009; Biro et al., 2010; Ogden et al., 2012).

Epidemiology studies suggest that age at onset of puberty in girls is decreasing over the years (Anderson and Must, 2005; Cesario and Hughes, 2007; Golub et al., 2008; Ahmed et al., 2009; Rosenfield et al., 2009; Biro et al., 2010; Burt Solorzano and McCartney, 2010); some propose that this decrease, as well as an increase in diagnosed cases of CPP in girls, would be associated to a cuncurrent rise in childhood obesity (Wattigney et al., 1999; Kaplowitz et al., 2001; Anderson et al., 2003; Lee et al., 2007; Kaplowitz, 2008). While there is positive correlation of obesity with early onset of puberty in some studies, this is not true for others. An example is a strictly controlled Copenhagen Puberty Study, in which early puberty remained significant after adjustment for body mass index (BMI) (Aksglaede et al., 2009). Another example is the study by Prete and cols, in which obesity was not a significantly contributor to premature puberty in the population studied (Prete et al., 2008).

The distinct ethnic distribution of ICPP reported in several studies emphasizes the genetic basis of this disorder. Additionally, these studies show that contribution of ethnicity to early puberty is significantly heavier than that of obesity (Anderson et al., 2003; Rosenfield et al., 2009; Biro et al., 2010; Walvoord, 2010; Ogden et al., 2012). Among girls of normal BMI, those of African American descent start puberty earlier than any other ethnicity (Biro et al., 2008; Rosenfield et al., 2009). Within the same ethnicity, a minority of overweight/obese girls develops ICPP: 72% of African American girls are overweight (BMI >25 and <30) or obese (BMI>30) but only 23% of these developed ICPP. Similarly, 61% of Hispanic girls are overweight or obese, but only 15% developed ICPP. Thirty-six percent white girls are overweight or obese but 10% developed ICPP (Biro et al., 2010; Ogden et al., 2012). Also, the last US National Health and Nutrition Evaluation Survey (NHANES) shows a clear contribution of obesity-independent genetic factors to early puberty (Rosenfield et al., 2009). This survey shows that obesity accelerates puberty only in early maturating girls, whereas thelarche or pubarche were not affected by obesity in late maturating girls (Rosenfield et al., 2009).

Lastly, obesity did not appear to have been a factor in the ICPP developed by the two children carrying the naturally occurring genetic mutations in KISS1R (Arg386Pro), which was identified in a girl (Teles et al., 2008) or in kisspeptin (Pro74Ser), which was identified in an unrelated toddler boy (Silveira et al., 2010).

Of note, correlation of obesity with puberty is sexually dimorphic as well. Rates of obesity are significantly higher in boys than in girls of pubertal age (age 6–11) in all ethnicities tested (White, Hispanic and African American) (Ogden et al., 2012). However, obesity in boys is largely associated to an opposed phenotype of delayed puberty and low testosterone, whereas early sexual maturation in boys is associated to lower rates of obesity when compared to later maturing boys (Wang, 2002; Burt Solorzano and McCartney, 2010; Walvoord, 2010).

## Naturally occurring genetic mutations in humans with ICPP

### Kisspeptin and kisspeptin receptor

A role for KISS1R and kisspeptin in the etiology of ICPP was revealed by the identification of two mutations with confirmed association to ICPP, one in KISS1R (Arg386Pro) and the other in kisspeptin (Pro74Ser) (Teles et al., 2008; Silveira et al., 2010). As opposed to KISS1R mutations associated to IHH; however, the two affected children carry the associated *gain-of-function* mutation in the *heterozygous* state. This is in conformity with the autosomal dominant inheritance (one mutated allele is enough for the manifestation of the phenotype) predicted for familial ICPP (de Vries et al., 2004). Nonetheless, tests performed for an additional kisspeptin mutant identified in two unrelated Brazilian girls (His90Asp) did not detect significant changes in the activity of the mutant (Silveira et al., 2010). Two additional mutations associated with ICPP in genome-wide association studies (His196Pro-KISS1R and Pro110Thr-kisspeptin) await confirmation of this association (Luan et al., 2002, 2007).

To date, mutations in KISS1R or kisspeptin appear to account for a minority of ICPP cases, as the majority of patients investigated to date have no mutations in the exons or exon-intron boundaries of *KISS1R* or *KISS1*.

### Mutations in other genes investigated in human ICPP

Among a plethora of proteins in which genetic mutations could potentially affect pubertal development, this section focus on genes that have been investigated in patients with ICPP, which include *TAC3*, *TACR3*, *LIN28B*, *GABRA1*, and *NPY*.

#### TACR3 and TAC3 genes

The *TACR3* encodes the G protein-coupled receptor neurokinin B (NKR3), and the *TAC3* gene encodes neurokinin B (NKB), the natural ligand for the NKR3. NKB and NKR3 are co-expressed with kisspeptin in a unique set of neurons named KNDy neurons, which are conserved across mammalian species and have been described for humans (Hrabovszky et al., 2010), monkeys (Ramaswamy et al., 2010), sheep (Goodman et al., 2007), goat (Wakabayashi et al., 2010), rat (Burke et al., 2006), and mice (Navarro et al., 2009). Increase in serum gonadotropins in response to stimulation of KNDy neurons by senktide (an NKB analog) has been demonstrated in monkeys (Ramaswamy et al., 2010), rats (Navarro et al., 2011), and ewes (follicular phase only) (Billings et al., 2010).

Loss-of-function mutations in NKB or in NK3R have recently implicated this ligand/receptor pair in the etiology of IHH in humans (Guran et al., 2009; Topaloglu et al., 2009; Fukami et al., 2010; Gianetti et al., 2010; Young et al., 2010; Francou et al., 2011). Also, one mutation in NK3R has been identified in a patient with ICPP (Ala63Pro-NK3R). However, association of this mutation with ICPP is yet to be confirmed, as the same mutation is present in the patient's mother, who reports normal pubertal development, and no functional assays have been performed for this mutation (Tusset, 2010; Teles et al., 2011).

#### LIN28B

*LIN28B* is the human homolog of a *C. elegans* gene with a role in timing larvae to adult maturation, which suggests that *LIN28B* could play a role in human sexual maturation. This is supported by genome-wide association studies indicating that polymorphisms in or near the *LIN28B* gene could be significant sources of variation in the age at menarche in girls (He et al., 2009; Ong et al., 2009; Perry et al., 2009). One mutation in *LIN28B* was indentified in the heterozygous state in a 4 year-old girl with sporadic (not inherited) ICPP. Nonetheless, functional assays performed did not detect significant changes in the activity of this mutant (Teles et al., 2011); thus, significance of *LIN28B* for human pubertal maturation remains unknown.

#### GABRA1

*GABRA1* encodes the gamma amino butyric acid A1 receptor α-1 subunit, which is reported to be essential for the effects of the gamma-aminobutyric acid type A (GABA_A_) receptors on GnRH neurons (Lee et al., 2010). An interest in investigating GABA_A_ receptors in girls with ICPP came from studies showing that a GABA_A_ receptor antagonist (bicuculine) accelerated puberty in monkeys (Keen et al., 1999), and that this effect was mediated by kisspeptin as indicated by robust increases in kisspeptin secretion in response to bicuculine (Kurian et al., 2012). Also, the effect of bicuculine on GnRH neurons was prevented by pre-treatment with anti-kisspeptin serum (Terasawa et al., 2010; Kurian et al., 2012). However, sequencing the *GABRA1* gene in a cohort of girls with ICPP did not detect mutations (Brito et al., 2006). Additionally, selective reduction of GABA_A_ receptors in GnRH neurons in mice did not result in visible pubertal abnormalities (Lee et al., 2010), suggesting that deficiencies in this receptor would be compensated for in rodents.

#### NPY receptor

The *NPYR* gene encodes the receptor for neuropeptide Y (NPY), which antagonizes GABA effects on GnRH neurons. This antagonism was shown to play a role in pubertal development in monkeys and rodents (Terasawa and Fernandez, 2001). Additionally, hypothalamic NPY-producing neurons were shown to co-express Kiss1r and respond to kisspeptin in mouse cells and sheep hypothalamic explants. These observations raised the possibility that mutations in the *NPYR* gene could play a role in the etiology of ICPP (Backholer et al., 2010; Kim et al., 2010). Nonetheless, sequencing of the NPY receptor-1 detected only a synonymous (does not result in amino acid substitution) single nucleotide polymorphism (SNP) in the heterozygous state in a girl with familial ICPP, and this polymorphism was present at a higher rate in the control population (28%). Moreover, *in vitro* assays failed to show altered activity for this mutant (Freitas et al., 2007).

## Kisspeptin receptor signaling and puberty

The KISS1R was first linked to reproductive function in 2003, when loss-of-function mutations in this receptor were associated to IHH in two unrelated consanguineous families (de Roux et al., 2003; Seminara et al., 2003). Affected members of both families carried the associated mutation in the homozygous state, whereas heterozygous siblings and parents had no obvious reproductive abnormalities (de Roux et al., 2003; Seminara et al., 2003). Additional loss-of-function mutations in *KISS1R* were subsequently identified in patients with IHH (de Roux et al., 2003; Lanfranco et al., 2005; Semple et al., 2005; Pallais et al., 2006; Tenenbaum-Rakover et al., 2007; Teles et al., 2010; Nimri et al., 2011). More recently, a loss-of-function mutation in the kisspeptin gene (*KISS1*) was also associated to IHH in a consanguineous family with history of IHH (Topaloglu et al., 2012). Similarly, disruption of *Kiss1r* or *Kiss1* in mice resulted in a phenotype compatible with that of IHH in humans (Funes et al., 2003; Seminara et al., 2003; Dungan et al., 2007; Kauffman et al., 2007b; Lapatto et al., 2007; Colledge, 2009). Conversely, kisspeptin treatment was shown to advance puberty in intact female mice (Navarro et al., 2004b), and two *gain-of-function* mutations (one in the *KISS1R* and the other in the *KISS1* gene) were identified in children with ICPP (Teles et al., 2008; Silveira et al., 2010). These observations validate the role of KISS1R and kisspeptin as essential regulators of GnRH secretion and onset of puberty.

### Kisspeptin and GnRH secretion

All loss-of-function mutations in *KISS1R* or *KISS1* have been shown or are predicted to impair G protein signaling by the KISS1R, which in turn blocks stimulation of GnRH secretion by this receptor, impairing spontaneous onset of puberty (de Roux et al., 2003; Lanfranco et al., 2005; Semple et al., 2005; Pallais et al., 2006; Tenenbaum-Rakover et al., 2007; Teles et al., 2010; Nimri et al., 2011). On the other hand, kisspeptin expression was shown to be high and to increase during puberty in the infundibular nucleus of the medium basal hypothalamus (MBH) in male and female Rhesus monkeys (Hrabovszky et al., 2010). This pubertal increase in kisspeptin is accompanied by parallel changes in GnRH pulses, which suggests a connection between the increase in kisspeptin and the pubertal changes in GnRH pulses (Shahab et al., 2005). The MBH is reported to contain the majority of neuroendocrine GnRH neurons in primates and in humans (Krey et al., 1975; Plant et al., 1978; Hrabovszky et al., 2010). Additional reports show pubertal increases in hypothalamic kisspeptin expression for mice (Herbison et al., 2010), rats (Navarro et al., 2004a,b), and teleost fish cobia (Mohamed et al., 2007), which demonstrates phylogenetic conservation of the effect of kisspeptin on sexual maturation across species. Accordingly, kisspeptin has been shown to stimulate gonadotropin secretion in humans (Dhillo et al., 2005), sheep (Messager et al., 2005), pigs (Lents et al., 2008), rats (Matsui et al., 2004; Navarro et al., 2005; Pheng et al., 2009), mice (Gottsch et al., 2004), and gilts (Lents et al., 2008). Pubertal increases in *Kiss1r* expression in GnRH neurons have also been reported in mice (Herbison et al., 2010).

The role of kisspeptin signaling on GnRH pulses is emphasized by the disruptive effect of the infusion of a kisspeptin antagonist (peptide 234) on GnRH pulses in Rhesus monkeys as well as on gonadotropin secretion in the ewe (Millar et al., 2010; Guerriero et al., 2012). The detection of Kiss1r expression in GnRH neurons of cichlid fish (Parhar et al., 2004), rats (Irwig et al., 2004), and mice (Herbison et al., 2010) further supports a role for kisspeptin/Kiss1r on GnRH secretion, as well as suggest that kisspeptin would activate GnRH secretion by direct bind to receptors on GnRH neurons. Localization of kisspeptin fibers in close apposition with GnRH neurons in mice suggests that Kiss1r would be expressed at both somata and dentrites (Wray and Gainer, 1987), enabling kisspeptin to activate signaling on somata and/or dentrites of GnRH neurons in mice. In humans, kisspeptin-containing axons have been reported to be in contact with dentrites of GnRH neurons (Hrabovszky et al., 2008).

In rodents, the arcuate and the anteroventral periventricular (AVPV) nuclei of the hypothalamus are believed to regulate GnRH pulsatility. The arcuate nucleus is the target of a potent negative feedback of estrogen on gonadotropin secretion, whereas the AVPV is the target of positive feedback of estrogen on gonadotropin secretion (Mayer et al., 2010). Interestingly, virtually all kisspeptin neurons of the AVPV and of the arcuate nucleus in mice coexpress estrogen receptor-α (ER-α). Disruption of ER-α on kisspeptin neurons abolishes both negative and positive effects of estrogen on gonadotropin secretion (Smith et al., 2005a, 2006b), which suggests that kisspeptin neurons mediate estrogenic effects on gonadotropin secretion (Smith et al., 2005a, 2006b). This is supported by the absence of estradiol or androgen receptors on GnRH neurons (Herbison and Theodosis, 1992; Huang and Harlan, 1993). On the other hand, kisspeptin neurons contain ER-α (Smith et al., 2005a,b; Franceschini et al., 2006), progesterone (Smith et al., 2007) and androgen (Smith et al., 2005b) receptors, and a slow release of estrogen restraint on kisspeptin neurons of the arcuate nucleus is reported to precede the pubertal increase in kisspeptin (Takumi et al., 2011).

## Sexual dimorphism of kisspeptin expression and association to reproductive function

Kisspeptin neurons, kisspeptin expression and/or serum kisspeptin have been consistently shown to be sexually dimorphic in many species, including humans (Wray and Gainer, 1987; Kauffman et al., 2007a,b; Homma et al., 2009; Kauffman et al., 2009; Bakker et al., 2010; Hrabovszky et al., 2010; Jayasena et al., 2011; Pita et al., 2011a). This dimorphism has been associated to the onset of puberty and fertility in some species (Wray and Gainer, 1987; Kauffman et al., 2007a,b; Homma et al., 2009; Kauffman et al., 2009; Bakker et al., 2010; Hrabovszky et al., 2010; Jayasena et al., 2011; Pita et al., 2011a). Prenatal exposure to sex steroids may account for at least part of the sexual dimorphism in kisspeptin, and lack of kisspeptin dimorphism can lead to irreversible abnormalities of the sexual behavior in some species (Kauffman et al., 2007a,b; Gonzalez-Martinez et al., 2008). Also, circulating kisspeptin has been reported to be sexually dimorphic in humans, with women having significantly elevated kisspeptin when compared to men (Wray and Gainer, 1987; Kauffman et al., 2007a, 2009; Hrabovszky et al., 2010; Pita et al., 2011a,b). Expression of the kisspeptin receptor has also been reported to be sexually dimorphic in rats (Navarro et al., 2004a), Rhesus monkeys (Shahab et al., 2005), and teleost fish cobia (Mohamed et al., 2007), which demonstrates phyllogenetic conservation of this effect as well.

Observations discussed below support an association between the sexual dimorphism in KISS1R/Kiss1r/kisspeptin and the sexual dimorphism of the onset of puberty and that of pubertal disorders.

### Relevance of the sexual dimorphism in kisspeptin for the reproductive function in rodents

The AVPV, which is implicated in the generation of the LH surge and ovulation in females (Smith et al., 2005b), has substantial sexual dimorphism of kisspeptin expression in rodents, with the male-typical expression pattern been established just before and after birth (Kauffman et al., 2007b). This corresponds to the timing of brain masculinization reported for mice. Kisspeptin fibers in the AVPV of sexually mature females is 12-fold elevated in rats (Kauffman et al., 2007a; Bakker et al., 2010) and ~15-fold elevated in mice (Wray and Gainer, 1987). On the other hand, kisspeptin expression in the arcuate nucleus of mice showed steroid-dependent sexual dimorphism only before puberty (Kauffman et al., 2009; Kauffman, 2010). After puberty, hypothalamic kisspeptin expression in both sexes responded similarly to changes in serum levels of sex steroids caused by gonadectomy (Kauffman et al., 2009). This juvenile distinction could be a sign of earlier onset of puberty in female mice, which would place rodents among species with a sexual dimorphism in pubertal development, in which females maturate earlier than males.

### Consequences of lack of kisspeptin sexual dimorphism in rodents

Disruption of the *Kiss1r* gene leads to loss of the sexual dimorphism in kisspeptin expression in the hypothalamus of male and female mice (Kauffman et al., 2007b). *Kiss1r* null females fail to ovulate and are infertile (Chan et al., 2009), whereas signs of abnormal sexual maturation in null males resemble those of male mice with aromatase insufficiency (Kauffman et al., 2007b; Bakker et al., 2010). Incidentally, the lack of brain masculinization in male and female mice with aromatase insufficiency has been attributed to the absence of masculinization of kisspeptin neurons, as indicated by inverse cFos activation in kisspeptin neurons of males and females in response to urinary odors in affected mice (Bakker et al., 2010).

Postnatal administration of testosterone rescued male copulatory behavior in aromatase-insufficient as well as in *Kiss1r* null male mice; however, female preference and other kisspeptin-dependent sexually dimorphic traits could not be recovered with postnatal sex steroid replacement in either animal model (Kauffman et al., 2007b; Bakker and Baum, 2008). These observations suggest that the sexual dimorphism in kisspeptin expression is critical for typical male/female sexual responses, which in turn implicates kisspeptin in the mediation of olfactory signals to reproduction (Bakker et al., 2010).

Abnormal sexual differentiation of the brain has been atributted to the abnormal sexual dimorphism in kisspeptin expression in other animal models such as α-fetoprotein-deficient mice (Gonzalez-Martinez et al., 2008). Placental α-fetoprotein metabolizes estrogens, which protects developing females from excessive exposure to maternal estrogens (Bakker et al., 2006). Masculinization of the female brain in α-fetoprotein-deficient females has been attributed to the altered sexual dimorphism of kisspeptin expression in the AVPV of affected females, who are infertile and incapable of generating LH surges in response to steroid stimulation (Gonzalez-Martinez et al., 2008). Similar masculinization of the female brain is described for neonatal female mice abnormally exposed to sex steroids, who exhibit “male-typical” low kisspeptin expression in the AVPV and an inability to generate LH surges (Kauffman et al., 2007a). Conversely, castration of neonatal male mice right after birth prevented brain masculinization. This resulted in males with female-typical kisspeptin expression in the AVPV, who exhibited an unusual ability of mounting LH surges in response to steroid stimulation (Homma et al., 2009).

### Serum kisspeptin and sexual dimorphism in humans

Recent studies have investigated expression and/or secretion of kisspeptin in adults as well as in pubertal children. While not definitive, the results of these studies consistently show sexually dimorphic differences in serum levels as well as in the expression of kisspeptin in humans. In one study, sexually dimorphic differences were identified in the distribution and number of immunolabeled kisspeptin in hypothalamic areas relevant for reproductive function in humans, with females exhibiting heavily labeled kisspeptin in the infundibulus, whereas very few, if any, were present in males (Hrabovszky et al., 2010). Brain samples analyzed in this study were obtained from healthy human subjects who died of sudden death (Hrabovszky et al., 2010). Results were confirmed with an additional antibody from a distinct source, the analysis was blinded, and the age of research subjects did not influence the results (Hrabovszky et al., 2010). The homogeneity of the data in the female group was reassuring against the potential variability of (unknown) sex steroid levels at the time of death among research subjects. Nonetheless, findings should be confirmed in brains from subjects with similar levels of sex steroids at the time of death.

Although the source of circulating kisspeptins has not been established, experimental studies show that intravenously injected kisspeptins can effectively stimulate GnRH/gonadotropin/steroid secretion in humans (Dhillo et al., 2005; George et al., 2011) and animal models such as Rhesus monkeys (Ramaswamy et al., 2007), rats (Matsui et al., 2004; Pheng et al., 2009), and mice (Mikkelsen et al., 2009). Systemic injections of physiologically relevant concentrations of kisspeptin synchronizes LH surge in cycling ewes and induces ovulation in non-cycling ewes on the anestrous season (Jayasena et al., 2010). Similarly, kisspeptin injected peripherally to women is capable of inducing desensitization of the LH response (Jayasena et al., 2009) as well as of bypassing the suppression of LH in patients affected with hypothalamic amenorrhea (Caraty et al., 2007). These observations demonstrate that circulating kisspeptins are physiologically relevant and likely to play a role in the regulation of the HPG axis in many species, including humans.

Serum kisspeptin in adult, sexually mature women was significantly elevated when compared to adult men of similar age in two studies of different populations from distinct ethnic backgrounds (Pita et al., 2011a,b). This endorses the sexually dimorphic character of kisspeptin differences, as opposed to other ethnic-specific genetic factors.

In healthy children, serum kisspeptin is reported to positively correlate with rises in LH and testosterone during all stages of puberty in boys (Bano et al., 2009). Likewise, serum kisspeptin in pubertal girls is reported to positively correlate to bone age, peak/basal LH, and LH/FSH ratios (Rhie et al., 2011). Additionally, healthy pubertal girls from an unrelated population were reported to have significantly elevated serum kisspeptin when compared to tanner grade-matched healthy boys, which are, in average, one year older (Pita et al., 2011a). These observations endorse serum kisspeptin as faithful indicator of onset and progression of puberty in healthy children as well as provide support for the involvement of kisspeptin in the mediation of the onset and progression of puberty in children.

## Kisspeptin as an integrator of nutritional, hormonal, and other signals to puberty—a hypothesis for the etiology of ICPP

### Leptin as the main mediator of nutritional signals to puberty

Fat-produced leptin is regarded as the main mediator of nutritional signals to reproduction. A role for leptin on the onset of puberty is evident in leptin-deficient *ob/ob* mice, which have arrested puberty and infertility (Swerdloff et al., 1976; Batt et al., 1982). Similar phenotype is observed in humans with congenital leptin deficiency, who present with hypogonadotropic hypogonadism and other symptoms of leptin deficiency such as hyperphagia and early onset obesity. All abnormalities are at least partially rescued with leptin supplementation in humans (Montague et al., 1977; Clement et al., 1998; Kiess et al., 1998; Strobel et al., 1998; Farooqi et al., 1999, 2002; Ozata et al., 1999; Gibson et al., 2004; Licinio et al., 2004) and in *ob/ob* mice (Halaas et al., 1995; Barash et al., 1996; Chehab et al., 1996; Mounzih et al., 1997; Kiess et al., 1998).

Co-regulation of reproductive function by nutrition is thought to improve species survival by suppressing reproduction during adversities such as negative energy balance. The absence of leptin in *ob/ob* mice leads to a genetically induced state of negative energy balance. A similar state of negative energy balance is associated with the suppression of serum leptin in intact humans and experimental animals subjected to fasting (Nagatani et al., 1998; Licinio et al., 2004; Welt et al., 2004). Negative energy balance with suppression of serum leptin is also associated with loss of body fat due to extreme exercise routines or eating disorders in humans (Licinio et al., 2004; Welt et al., 2004). The negative energy balance in these cases is also reversed with leptin supplementation (Licinio et al., 2004; Welt et al., 2004).

While there is no doubt that leptin plays a role in the onset of puberty, current evidence is not enough to define the precise nature of this effect. Some argue that the initiation of puberty in humans (Frisch and Revelle, 1970) and rodents (Kennedy and Mitra, 1963) would require a critical fat mass, and that the resulting increase in fat-produced leptin would be the signal to initiate puberty once this critical fat mass is achieved (Barash et al., 1996; Chehab et al., 1996). The fact that injection of leptin accelerates puberty in normal female mice would support the requirement of a critical fat mass for the onset of puberty (Ahima et al., 1997; Chehab et al., 1997). However, Cheung and cols (Cheung et al., 2001) found unchanged pre-pubertal and pubertal serum leptin in male and female rats, and serum leptin only correlated with body weight *after* puberty in all tested animals (Cheung et al., 2001).

A similar pattern is observed in children. The three largest epidemiology studies in children show that leptin levels are similar in pre-pubertal boys and girls. Sexually dimorphic differences in leptin only appear at later stages of pubertal development, when boys and girls are at tanner grades 2–5. At the late stages, girls exhibit elevated serum leptin when compared to boys. This increase in serum leptin in females could be attributed to the pubertal rise in circulating estradiol, as clinical data shows that estrogen increases leptin in women independently of body fat content (Lavoie et al., 1999). After correction for fat mass, women have higher serum leptin than men; premenopausal women have higher leptin than postmenopausal women, and short-term estrogen replacement increases serum leptin in postmenopausal women independently of changes in fat mass. This effect is estrogen-specific, as progesterone replacement did not affect serum leptin (Lavoie et al., 1999). These observations suggest that the pubertal increase in leptin could be a consequence (rather than the trigger) of puberty in healthy children (Blum et al., 1997; Clayton et al., 1997; Ahmed et al., 1999).

Additional clinical evidence demonstrates that, despite undetectable *serum* leptin, patients from a family with history of lipoatropic diabetes who have virtually no subcutaneous or visceral fat have normal sexual maturation and menarche (Andreelli et al., 2000). Despite severe leptin deficiency, serum gonadotropins, gonadal steroids, subsequent menstrual cycles, and fertility were not affected in these patients (Andreelli et al., 2000). These phenotypic characteristics are in contradiction to those of patients homozygous for mutations that inactivate leptin or the leptin receptor, which develop early onset obesity and IHH. This inconsistency exposes a gap in our knowledge of how leptin regulates energy metabolism and reproductive function. Nonetheless, the explanation for the inconsistencies may lie in one fundamental difference: despite suppressed, any endogenous leptin produced in patients with lipoatropic diabetes is biologically active, whereas patients with homozygous loss-of-function mutations in leptin or the leptin receptor are simply incapable of activating leptin receptor signaling.

### Kisspeptin as the main target of leptin signals to reproductive function

Kisspeptin is a recognized target of leptin (Smith et al., 2006a; Backholer et al., 2010). In fact, kisspeptin is believed to be the main mediator of pubertal effects of leptin. Kisspeptin expression is highly sensitive to variations in serum leptin or in the nutritional state (Castellano et al., 2005; Smith et al., 2006a; Luque et al., 2007; Kalamatianos et al., 2008; Roa et al., 2008; Quennell et al., 2011), which is compatible with the involvement of kisspeptin in the transmission of nutritional signals to reproduction. Accordingly, expression of kisspeptin is suppressed in animal models of congenital leptin deficiency or negative energy balance (Castellano et al., 2005; Luque et al., 2007; Kalamatianos et al., 2008), and kisspeptin administration increases gonadotropin secretion in leptin-deficient or fasted rodents (Navarro et al., 2004b; Castellano et al., 2005; Roa et al., 2008). In humans, kisspeptin administration has been shown to counteract the suppression of serum gonadotropins associated with hypothalamic amenorrhea, a human model of negative energy balance (Caraty et al., 2007).

Interestingly, the above effects of kisspeptin occur in spite of the unaltered state of negative energy balance and suppression of serum leptin, as well as the absence of changes in body weight or food intake in affected women or experimental animals. This indicates that kisspeptin is able to bypass the negative state of energy balance to activate the HPG axis, suggesting that stimulation of the reproductive axis by kisspeptin is downstream of metabolic signals such as leptin. This is further supported by the rescue of vaginal opening, serum gonadotropins, and estradiol in fasted female mice treated with kisspeptin (Castellano et al., 2005). Moreover, administration of kisspeptin accelerates puberty in pre-pubertal female rodents despite prior treatment with anti-leptin antibody or negative state of energy balance due to fasting or leptin-resistance (Navarro et al., 2004b, 2005; Castellano et al., 2005).

In rodents, leptin effects on puberty have been presumed to result from binding of leptin to receptors located in kisspeptin neurons of the arcuate nucleus. However, a study using mice with selective ablation of leptin receptors in kisspeptin neurons is challenging this presumption. Ablated mice had normal pubertal development and were fertile (Donato et al., 2011). Additionally, lesions targeting neurons within the hypothalamic ventral premammillary nucleus (PMV) blocked the progression of puberty in *ob/ob* mice by exogenous leptin. These observations are against the requirement of a direct effect of leptin on kisspeptin neurons. They also suggest that the main target of leptin would not be kisspeptin neurons of the arcuate nucleus. Instead, reproductive effects of leptin would be mediated through the PMV (Donato et al., 2011). These observations challenge our perception of how leptin stimulates puberty, and suggest an indirect effect of leptin on kisspeptin, which would be triggered by binding of leptin to receptors within the PMV (rather than in the arcuate) nucleus. This is supported by the absence of leptin receptors in the majority of kisspeptin neurons in the arcuate nucleus (Herbison et al., 2010) as well as by observations that fasting suppresses kisspeptin expression in the AVPV (which do not express leptin receptors) but not in the arcuate nucleus in rats (Kalamatianos et al., 2008). Nonetheless, current experimental and clinical data consistently point to kisspeptin as (indirect?) mediator of leptin signals to reproduction.

### Proposed hypothesis

**Figure 1:**
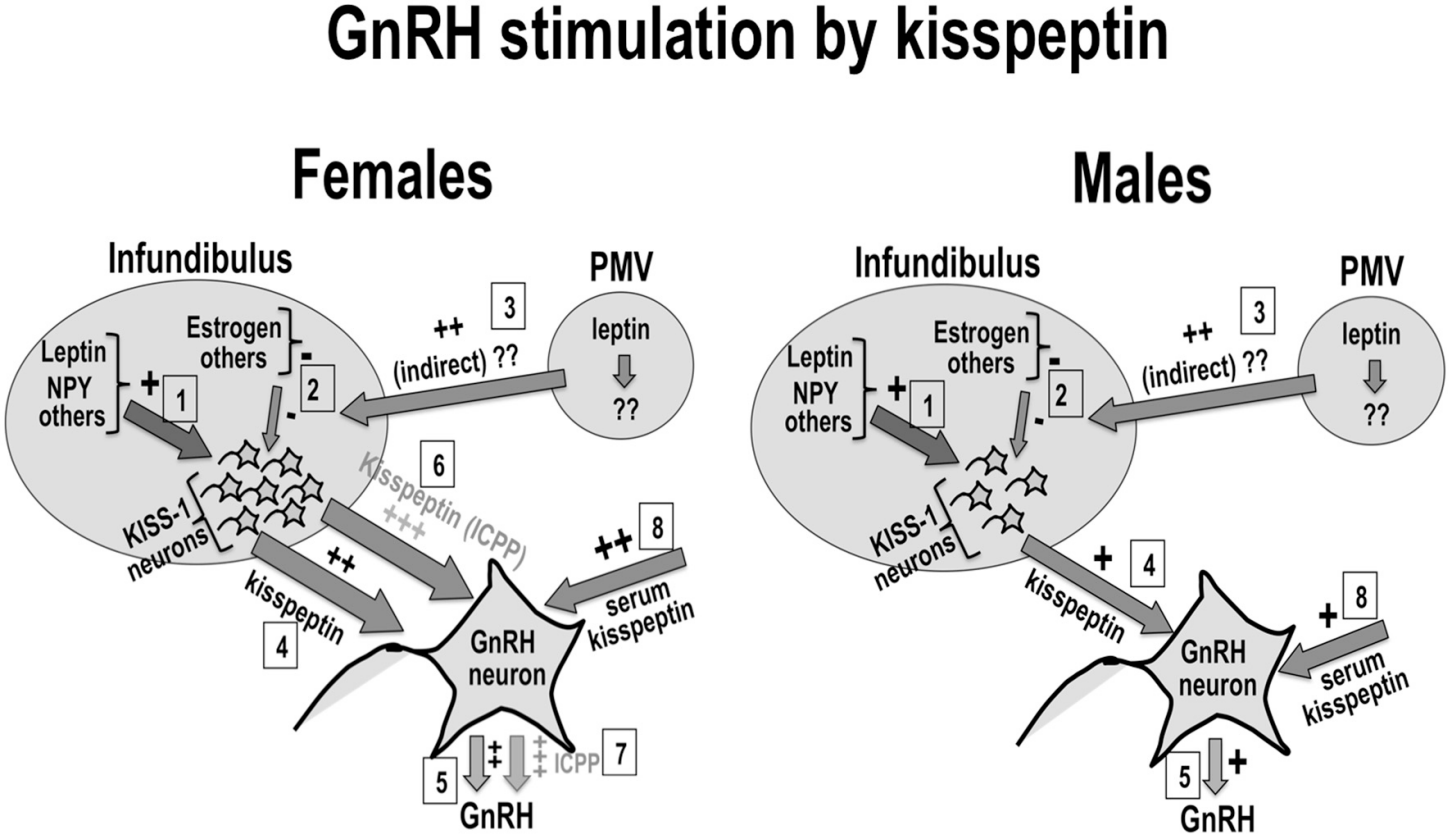
**Kisspeptin, sexual dimorphism, and puberty: Kisspeptin neurons (KISS-1) in the infundibular nucleus would be regulated by positive (1) and negative (2) inputs from nutritional, hormonal and environmental sources.** Possible leptin input on kisspeptin neurons is shown in (1) and (3). Kisspeptin from KISS-1 neurons (4) or the circulation (8) stimulate GnRH neurons to secrete GnRH (5). The number of KISS-1 neurons in the infundibulus is reported to be elevated in women (left panel) when compared to men (right panel). This sexual difference in kisspeptin is proposed to contribute to earlier onset of puberty in healthy girls when compared to boys (6), as well as to the higher incidence of idiopathic CPP in girls (7).

The sexual dimorphism in the distribution of kisspeptin neurons, in kisspeptin expression, and/or serum kisspeptin is proposed to contribute to the sexual dimorphism in the onset of puberty reported for humans and experimental animals. Additionally, the effect of kisspeptin on the onset and progression of puberty is proposed to depend on the contribution of puberty signals from nutritional, hormonal, environmental, and other sources, which would be integrated at the kisspeptin level in order to customize/optimize reproductive function. Figure 1 shows a schematic representation of this hypothesis, in which pubertal stimuli would converge into kisspeptin neurons to fine-tune the effect of these on GnRH stimulation by kisspeptin. Figure 1 also shows the proposed effects on girls and boys of progressive increases in kisspeptin secretion along puberty. A hypothetically elevated number of kisspeptin fibers is proposed to contribute to the earlier onset of puberty in girls when compared to age-matched boys. This is also proposed to contribute to the significantly elevated incidence of CPP in girls.

The above hypothesis is supported by the sexual dimorphism in serum kisspeptin reported for pre-pubertal girls (tanner grade 1), who exhibit elevated serum kisspeptin when compared to age- or tanner grade-matched boys (Pita et al., 2011a). This sexual dimorphism persists in adulthood and is corroborated by elevated expression of kisspeptin in the hypothalamus, which is reported for females of many species, including humans (Wray and Gainer, 1987; Cheng et al., 2010; Pita et al., 2011a,b). The sexual dimorphism in kisspeptin could be associated to the reported sexual dimorphism in gonadotropin secretion in humans. Serum gonadotropins are elevated in healthy, pre-pubertal girls (tanner grade 1) when compared to age- or tanner grade-matched boys (Nottelmann et al., 1987). Notably, boys in tanner grade 1 are in average one year older than tanner grade-matched girls.

A role for the sexual dimorphism in kisspeptin in the etiology of ICPP is supported by the elevated serum kisspeptin reported for girls with ICPP when compared to age-matched healthy girls^1^ (de Vries et al., 2009; Chen et al., 2010a; Rhie et al., 2011). These studies selected precocious girls from distinct ethnic backgrounds presenting with classic idiopathic CPP (advanced bone age and elevated LH and FSH peak responses) after other known causes of the disorder were discarded. Serum kisspeptin in precocious girls positively correlated with peak LH and LH/FSH ratio but not with BMI (de Vries et al., 2009; Rhie et al., 2011). In one study, serum kisspeptin in girls with ICPP correlated with urinary levels of monobutyl phthalate, suggesting that acceleration of puberty induced by this endocrine disruptor could be mediated by kisspeptin (Chen et al., 2010a).

The involvement of kisspeptin in the etiology of ICPP is also supported by two mutations with confirmed association to the disorder, which were identified in kisspeptin (Pro74Ser) or the KISS1R (Arg386Pro). Of note, both mutations lead to surprisingly modest increases in signaling, and require KISS1R activation by kisspeptin, which demonstrates that these mutants lack constitutive activation. The small magnitude of the effects of these mutants is presumed to be the basis for the *gain-of-function*, which for both mutants is due to a prolongation of the *endogenous* stimulation of KISS1R by kisspeptin (Teles et al., 2008; Silveira et al., 2010).

## Final considerations

Precocious or late puberty have implications for affected children, often requiring long-term counseling. Additionally, both disorders are associated with increased risk of other diseases. Early puberty is associated to obesity, polycystic ovaries, metabolic syndrome (for girls), and a variety of cancers (Deardorff et al., 2005; Franks, 2008; Golub et al., 2008; Burt Solorzano and McCartney, 2010; Franceschi et al., 2010), whereas late puberty is associated with metabolic syndrome (for boys), osteoporosis and osteoporotic fractures later in life (Finkelstein et al., 1992; Francis, 1999; Golub et al., 2008). New efficient strategies for early detection and prevention of pubertal disorders and their associated health risks require the understanding of the pathophysiological mechanisms underlying normal and abnormal puberty. For that, the identification and characterization of upstream regulators of GnRH pulsatility is of upmost importance.

One additional study comparing serum kisspeptin in pre-pubertal obese, pre-pubertal age-matched (normal), and ICPP girls found increased serum kisspeptin in the obese but not in the ICPP group (Pita et al., 2011a). A likely source of discrepancy with results of studies previously discussed here is the high cut-off peak LH adopted for the selection of the ICPP group: premature girls with peak LH <7.0 IU/L were excluded, whereas above studies excluded girls with peak LH <5.0U/L (de Vries et al., 2009) or did not adopt a LH cut-off value to exclude potential subjects (Rhie et al., 2011). Incidentally, the girl carrying the first genetic mutation with confirmed association to ICPP (Arg386Pro-KISS1R) would have been eliminated from this but not the other studies, as her peak LH was 6.4 IU/L (Teles et al., 2008). Additional support against the exclusion of girls with LH <7.0 IU/L is provided by the six-month follow-up of girls in the previous studies, which found no differences in kisspeptin or any other clinical, laboratorial or tanner stage parameters between ICPP girls with a peak LH >5.0 IU/l and those with a peak LH <5.0 IU/l (de Vries et al., 2009). Thus, the high LH cut-off value likely eliminated girls with true ICPP from the study by Pita and cols.

Further characterization of the complex relationship of reproductive function with energy metabolism may uncover the basis for inconsistencies such as the opposite effect of obesity on pubertal maturation in boys and girls (Wang, 2002; Burt Solorzano and McCartney, 2010; Walvoord, 2010), and the intriguing correlation of serum leptin with nocturnal but not with diurnal gonadotropin secretion in pubertal girls and adult women (Matkovic et al., 1997; Licinio et al., 1998). Equally intriguing is the inverse correlation of nocturnal serum leptin with weight gain in pubertal girls (Matkovic et al., 1997), and the puzzling phenotype of patients with lipoatropic diabetes, who have normal puberty despite the undetectable serum leptin. Investigation of serum kisspeptin during puberty and adulthood in these patients may provide mechanistic insights into phenotypic inconsistencies between these patients and those carrying homozygous loss-of-function mutations in leptin or leptin receptor.

Finally, despite the failure to identify genetic mutations in receptors for neurotransmitter such as NPY and GABA in ICPP girls, the possibility of the involvement of these or other receptors and signaling pathways in the etiology of ICPP cannot be discarded.

### Conflict of interest statement

The author declares that the research was conducted in the absence of any commercial or financial relationships that could be construed as a potential conflict of interest.

## Footnotes

^1^Relevance of serum kisspeptin to reproductive function is discussed in the second paragraph of section “Consequences of Lack of Kisspeptin Sexual Dimorphism in Rodents.”

## References

[B1] Ahima R. S. Dushay J. Flier S. N. Prabakaran D. Flier J. S. (1997). Leptin accelerates the onset of puberty in normal female mice. J. Clin. Invest. 99, 391–395 10.1172/JCI119172 9022071 PMC507811

[B2] Ahmed M. L. Ong K. K. Dunger D. B. (2009). Childhood obesity and the timing of puberty. Trends Endocrinol. Metab. 20, 237–242 10.1016/j.tem.2009.02.004 19541497

[B3] Ahmed M. L. Ong K. K. Morrell D. J. Cox L. Drayer N. Perry L. (1999). Longitudinal study of leptin concentrations during puberty: sex differences and relationship to changes in body composition. J. Clin. Endocrinol. Metab. 84, 899–905 10.1210/jc.84.3.899 10084568

[B4] Aksglaede L. Sorensen K. Petersen J. H. Skakkebaek N. E. Juul A. (2009). Recent decline in age at breast development: the Copenhagen Puberty Study. Pediatrics 123, e932–e939 10.1542/peds.2008-2491 19403485

[B5] Anderson S. E. Dallal G. E. Must A. (2003). Relative weight and race influence average age at menarche: results from two nationally representative surveys of US girls studied 25 years apart. Pediatrics 111, 844–850 12671122 10.1542/peds.111.4.844

[B6] Anderson S. E. Must A. (2005). Interpreting the continued decline in the average age at menarche: results from two nationally representative surveys of U.S. girls studied 10 years apart. J. Pediatr. 147, 753–760 10.1016/j.jpeds.2005.07.016 16356426

[B7] Andreelli F. Hanaire-Broutin H. Laville M. Tauber J. P. Riou J. P. Thivolet C. (2000). Normal reproductive function in leptin-deficient patients with lipoatropic diabetes. J. Clin. Endocrinol. Metab. 85, 715–719 10.1210/jc.85.2.715 10690881

[B8] Apter D. Pakarinen A. Vihko R. (1978). Serum prolactin, FSH and LH during puberty in girls and boys. Acta Paediatr. Scand. 67, 417–423 209664 10.1111/j.1651-2227.1978.tb16348.x

[B9] Backholer K. Smith J. T. Rao A. Pereira A. Iqbal J. Ogawa S. (2010). Kisspeptin cells in the ewe brain respond to leptin and communicate with neuropeptide Y and proopiomelanocortin cells. Endocrinology 151, 2233–2243 10.1210/en.2009-1190 20207832

[B10] Bakker J. Baum M. J. (2008). Role for estradiol in female-typical brain and behavioral sexual differentiation. Front. Neuroendocrinol. 29:1 10.1016/j.yfrne.2007.06.001 17720235 PMC2373265

[B11] Bakker J. De Mees C. Douhard Q. Balthazart J. Gabant P. Szpirer J. (2006). Alpha-fetoprotein protects the developing female mouse brain from masculinization and defeminization by estrogens. Nat. Neurosci. 9, 220–226 10.1038/nn1624 16388309

[B12] Bakker J. Pierman S. Gonzalez-Martinez D. (2010). Effects of aromatase mutation (ArKO) on the sexual differentiation of kisspeptin neuronal numbers and their activation by same versus opposite sex urinary pheromones. Horm. Behav. 57, 390–395 10.1016/j.yhbeh.2009.11.005 19945459

[B13] Bano R. Wahab F. F. Riaz T. Jabeen S. Irfan S. Arslan M. (2009). Peripheral metastin (kisspeptin 54) levels change during progression of puberty in boys in The 91th Endocrine Society Meeting (Washington, DC), 287, P3

[B14] Barash I. A. Cheung C. C. Weigle D. S. Ren H. Kabigting E. B. Kuijper J. L. (1996). Leptin is a metabolic signal to the reproductive system. Endocrinology 137, 3144–3147 10.1210/en.137.7.3144 8770941

[B15] Batt R. A. Everard D. M. Gillies G. Wilkinson M. Wilson C. A. Yeo T. A. (1982). Investigation into the hypogonadism of the obese mouse (genotype ob/ob). J. Reprod. Fertil. 64, 363–371 10.1530/jrf.0.0640363 6175745

[B16] Bedecarrats G. Y. Kaiser U. B. (2007). Mutations in the human gonadotropin-releasing hormone receptor: insights into receptor biology and function. Semin. Reprod. Med. 25, 368–378 10.1055/s-2007-984743 17710733

[B17] Bhagavath B. Podolsky R. H. Ozata M. Bolu E. Bick D. P. Kulharya A. (2006). Clinical and molecular characterization of a large sample of patients with hypogonadotropic hypogonadism. Fertil. Steril. 85, 706–713 10.1016/j.fertnstert.2005.08.044 16500342

[B18] Bianco S. D. Kaiser U. B. (2009). The genetic and molecular basis of idiopathic hypogonadotropic hypogonadism. Nat. Rev. Endocrinol. 5, 569–576 10.1038/nrendo.2009.177 19707180 PMC2864719

[B19] Bianco S. D. Vandepas L. Correa-Medina M. Gereben B. Mukherjee A. Kuohung W. (2011). KISS1R intracellular trafficking and degradation: effect of the Arg386Pro disease-associated mutation. Endocrinology 152, 1616–1626 10.1210/en.2010-0903 21285314 PMC3060635

[B20] Billings H. J. Connors J. M. Altman S. N. Hileman S. M. Holaskova I. Lehman M. N. (2010). Neurokinin B acts via the neurokinin-3 receptor in the retrochiasmatic area to stimulate luteinizing hormone secretion in sheep. Endocrinology 151, 3836–3846 10.1210/en.2010-0174 20519368 PMC2940514

[B21] Biro F. M. Galvez M. P. Greenspan L. C. Succop P. A. Vangeepuram N. Pinney S. M. (2010). Pubertal assessment method and baseline characteristics in a mixed longitudinal study of girls. Pediatrics 126, E583–E590 10.1542/peds.2009-3079 20696727 PMC4460992

[B22] Biro F. M. Huang B. Daniels S. R. Lucky A. W. (2008). Pubarche as well as thelarche may be a marker for the onset of puberty. J. Pediatr. Adolesc. Gynecol. 21, 323–328 10.1016/j.jpag.2007.09.008 19064225 PMC3576862

[B23] Blum W. F. Englaro P. Hanitsch S. Juul A. Hertel N. T. Muller J. (1997). Plasma leptin levels in healthy children and adolescents: dependence on body mass index, body fat mass, gender, pubertal stage, and testosterone. J. Clin. Endocrinol. Metab. 82, 2904–2910 9284717 10.1210/jcem.82.9.4251

[B24] Brito V. N. Mendonca B. B. Guilhoto L. M. Freitas K. C. Arnhold I. J. Latronico A. C. (2006). Allelic variants of the gamma-aminobutyric acid-A receptor alpha1-subunit gene (GABRA1) are not associated with idiopathic gonadotropin-dependent precocious puberty in girls with and without electroencephalographic abnormalities. J. Clin. Endocrinol. Metab. 91, 2432–2436 10.1210/jc.2005-2657 16569738

[B25] Burke M. C. Letts P. A. Krajewski S. J. Rance N. E. (2006). Coexpression of dynorphin and neurokinin B immunoreactivity in the rat hypothalamus: morphologic evidence of interrelated function within the arcuate nucleus. J. Comp. Neurol. 498, 712–726 10.1002/cne.21086 16917850

[B26] Burt Solorzano C. M. McCartney C. R. (2010). Obesity and the pubertal transition in girls and boys. Reproduction 140, 399–410 10.1530/REP-10-0119 20802107 PMC2931339

[B27] Caraty A. Smith J. T. Lomet D. Ben Said S. Morrissey A. Cognie J. (2007). Kisspeptin synchronizes preovulatory surges in cyclical ewes and causes ovulation in seasonally acyclic ewes. Endocrinology 148, 5258–5267 10.1210/en.2007-0554 17702853

[B28] Castellano J. M. Navarro V. M. Fernandez-Fernandez R. Nogueiras R. Tovar S. Roa J. (2005). Changes in hypothalamic KiSS-1 system and restoration of pubertal activation of the reproductive axis by kisspeptin in undernutrition. Endocrinology 146, 3917–3925 10.1210/en.2005-0337 15932928

[B29] Cesario S. K. Hughes L. A. (2007). Precocious puberty: a comprehensive review of literature. J. Obstet. Gynecol. Neonatal. Nurs. 36, 263–274 10.1111/j.1552-6909.2007.00145.x 17489932

[B30] Chan Y. M. Broder-Fingert S. Seminara S. B. (2009). Reproductive functions of kisspeptin and Gpr54 across the life cycle of mice and men. Peptides 30, 42–48 10.1016/j.peptides.2008.06.015 18644412 PMC2656499

[B31] Chehab F. F. Lim M. E. Lu R. (1996). Correction of the sterility defect in homozygous obese female mice by treatment with the human recombinant leptin. Nat. Genet. 12, 318–320 10.1038/ng0396-318 8589726

[B32] Chehab F. F. Mounzih K. Lu R. Lim M. E. (1997). Early onset of reproductive function in normal female mice treated with leptin. Science 275, 88–90 10.1126/science.275.5296.88 8974400

[B33] Chen C.-Y. Wu Y.-M. Chou Y.-Y. Lin S.-J. Lee C.-C. (2010a). Phthalate exposure may affect girls puberty via stimulation of kisspeptin-54 secretion, in Epidemiology (Ed.), ISEE 22ndAnnual Conference (Seoul, Korea: Lippincott Williams and Wilkins), S127

[B34] Chen X. Mo Y. Li L. Chen Y. Li Y. Yang D. (2010b). Increased plasma metastin levels in adolescent women with polycystic ovary syndrome. J. Eur. Obstet. Gynecol. Reprod. Biol. 149, 72–76 10.1016/j.ejogrb.2009.11.018 20022159

[B35] Cheng G. Coolen L. M. Padmanabhan V. Goodman R. L. Lehman M. N. (2010). The kisspeptin/neurokinin B/dynorphin (KNDy) cell population of the arcuate nucleus: sex differences and effects of prenatal testosterone in sheep. Endocrinology 151, 301–311 10.1210/en.2009-0541 19880810 PMC2803147

[B36] Cheung C. C. Thornton J. E. Nurani S. D. Clifton D. K. Steiner R. A. (2001). A reassessment of leptin's role in triggering the onset of puberty in the rat and mouse. Neuroendocrinology 74, 12–21 11435754 10.1159/000054666

[B37] Clarkson J. Herbison A. E. (2006). Postnatal development of kisspeptin neurons in mouse hypothalamus; sexual dimorphism and projections to gonadotropin-releasing hormone neurons. Endocrinology 147, 5817–5825 10.1210/en.2006-0787 16959837 PMC6098691

[B38] Clayton P. E. Gill M. S. Hall C. M. Tillmann V. Whatmore A. J. Price D. A. (1997). Serum leptin through childhood and adolescence. Clin. Endocrinol. (Oxf.) 46, 727–733 9274704 10.1046/j.1365-2265.1997.2081026.x

[B39] Clement K. Vaisse C. Lahlou N. Cabrol S. Pelloux V. Cassuto D. (1998). A mutation in the human leptin receptor gene causes obesity and pituitary dysfunction. Nature 392, 398–401 10.1038/32911 9537324

[B40] Cohen-Bendahan C. C. van de Beek C. Berenbaum S. A. (2005). Prenatal sex hormone effects on child and adult sex-typed behavior: methods and findings. Neurosci. Biobehav. Rev. 29, 353–384 10.1016/j.neubiorev.2004.11.004 15811504

[B41] Colledge W. H. (2009). Transgenic mouse models to study Gpr54/kisspeptin physiology. Peptides 30, 34–41 10.1016/j.peptides.2008.05.006 18571287

[B42] Deardorff J. Gonzales N. A. Christopher F. S. Roosa M. W. Millsap R. E. (2005). Early puberty and adolescent pregnancy: the influence of alcohol use. Pediatrics 116. 1451–1456 10.1542/peds.2005-0542 16322170

[B43] Deladoey J. Fluck C. Bex M. Yoshimura N. Harada N. Mullis P. E. (1999). Aromatase deficiency caused by a novel P450arom gene mutation: impact of absent estrogen production on serum gonadotropin concentration in a boy. J. Clin. Endocrinol. Metab. 84, 4050–4054 10.1210/jc.84.11.4050 10566648

[B44] Delemarre-van de Waal H. A. (2002). Regulation of puberty. Best Pract. Res. Clin. Endocrinol. Metab. 16, 1–12 10.1053/beem.2001.0176 11987894

[B45] de Roux N. Genin E. Carel J. C. Matsuda F. Chaussain J. L. Milgrom E. (2003). Hypogonadotropic hypogonadism due to loss of function of the KiSS1-derived peptide receptor GPR54. Proc. Natl. Acad. Sci. U.S.A. 100, 10972–10976 10.1073/pnas.1834399100 12944565 PMC196911

[B46] de Vries L. Kauschansky A. Shohat M. Phillip M. (2004). Familial central precocious puberty suggests autosomal dominant inheritance. J. Clin. Endocrinol. Metab. 89, 1794–1800 10.1210/jc.2003-030361 15070947

[B47] de Vries L. Shtaif B. Phillip M. Gat-Yablonski G. (2009). Kisspeptin serum levels in girls with central precocious puberty. Clin. Endocrinol. (Oxf.) 71, 524–528 10.1111/j.1365-2265.2009.03575.x 19508611

[B48] Dhillo W. S. Chaudhri O. B. Patterson M. Thompson E. L. Murphy K. G. Badman M. K. (2005). Kisspeptin-54 stimulates the hypothalamic-pituitary gonadal axis in human males. J. Clin. Endocrinol. Metab. 90, 6609–6615 10.1210/jc.2005-1468 16174713

[B49] Donato J. Jr. Cravo R. M. Frazao R. Gautron L. Scott M. M. Lachey J. (2011). Leptin's effect on puberty in mice is relayed by the ventral premammillary nucleus and does not require signaling in Kiss1 neurons. J. Clin. Invest. 121, 355–368 10.1172/JCI45106 21183787 PMC3007164

[B50] Dungan H. M. Gottsch M. L. Zeng H. Gragerov A. Bergmann J. E. Vassilatis D. K. (2007). The role of kisspeptin-GPR54 signaling in the tonic regulation and surge release of gonadotropin-releasing hormone/luteinizing hormone. J. Neurosci. 27, 12088–12095 10.1523/JNEUROSCI.2748-07.2007 17978050 PMC6673361

[B51] Farooqi I. S. Jebb S. A. Langmack G. Lawrence E. Cheetham C. H. Prentice A. M. (1999). Effects of recombinant leptin therapy in a child with congenital leptin deficiency. N. Engl. J. Med. 341, 879–884 10.1056/NEJM199909163411204 10486419

[B52] Farooqi I. S. Matarese G. Lord G. M. Keogh J. M. Lawrence E. Agwu C. (2002). Beneficial effects of leptin on obesity, T cell hyporesponsiveness, and neuroendocrine/metabolic dysfunction of human congenital leptin deficiency. J. Clin. Invest. 110, 1093–1103 10.1172/JCI15693 12393845 PMC150795

[B53] Finkelstein J. S. Neer R. M. Biller B. M. Crawford J. D. Klibanski A. (1992). Osteopenia in men with a history of delayed puberty. N. Engl. J. Med. 326, 600–604 10.1056/NEJM199202273260904 1734250

[B54] Franceschi R. Gaudino R. Marcolongo A. Gallo M. C. Rossi L. Antoniazzi F. (2010). Prevalence of polycystic ovary syndrome in young women who had idiopathic central precocious puberty. Fertil. Steril. 93, 1185–1191 10.1016/j.fertnstert.2008.11.016 19135667

[B55] Franceschini I. Lomet D. Cateau M. Delsol G. Tillet Y. Caraty A. (2006). Kisspeptin immunoreactive cells of the ovine preoptic area and arcuate nucleus co-express estrogen receptor alpha. Neurosci. Lett. 401, 225–230 10.1016/j.neulet.2006.03.039 16621281

[B56] Francis R. M. (1999). The effects of testosterone on osteoporosis in men. Clin. Endocrinol. (Oxf.) 50, 411–414 10.1046/j.1365-2265.1999.00730.x 10468898

[B57] Francou B. Bouligand J. Voican A. Amazit L. Trabado S. Fagart J. (2011). Normosmic congenital hypogonadotropic hypogonadism due to TAC3/TACR3 mutations: characterization of neuroendocrine phenotypes and novel mutations. PLoS ONE 6:e25614 10.1371/journal.pone.0025614 22031817 PMC3198730

[B58] Franks S. (2008). Polycystic ovary syndrome in adolescents. Int. J. Obes. (Lond.) 32, 1035–1041 10.1038/ijo.2008.61 18458678

[B59] Freitas K. C. Ryan G. Brito V. N. Tao Y. X. Costa E. M. Mendonca B. B. (2007). Molecular analysis of the neuropeptide Y1 receptor gene in human idiopathic gonadotropin-dependent precocious puberty and isolated hypogonadotropic hypogonadism. Fertil. Steril. 87, 627–634 10.1016/j.fertnstert.2006.07.1519 17140570

[B60] Frisch R. E. Revelle R. (1970). Height and weight at menarche and a hypothesis of critical body weights and adolescent events. Science 169, 397–399 10.1126/science.169.3943.397 5450378

[B61] Fukami M. Maruyama T. Dateki S. Sato N. Yoshimura Y. Ogata T. (2010). Hypothalamic dysfunction in a female with isolated hypogonadotropic hypogonadism and compound heterozygous TACR3 mutations and clinical manifestation in her heterozygous mother. Horm. Res. Paediatr. 73, 477–481 10.1159/000313373 20395662

[B62] Funes S. Hedrick J. A. Vassileva G. Markowitz L. Abbondanzo S. Golovko A. (2003). The KiSS-1 receptor GPR54 is essential for the development of the murine reproductive system. Biochem. Biophys. Res. Commun. 312, 1357–1363 10.1016/j.bbrc.2003.11.066 14652023

[B63] George J. T. Veldhuis J. D. Roseweir A. K. Newton C. L. Faccenda E. Millar R. P. (2011). Kisspeptin-10 is a potent stimulator of LH and increases pulse frequency in men. J. Clin. Endocrinol. Metab. 96, E1228–E1236 10.1210/jc.2011-0089 21632807 PMC3380939

[B64] Gianetti E. Tusset C. Noel S. D. Au M. G. Dwyer A. A. Hughes V. A. (2010). TAC3/TACR3 mutations reveal preferential activation of gonadotropin-releasing hormone release by neurokinin B in neonatal life followed by reversal in adulthood. J. Clin. Endocrinol. Metab. 95, 2857–2867 10.1210/jc.2009-2320 20332248 PMC2902066

[B65] Gibson W. T. Farooqi I. S. Moreau M. DePaoli A. M. Lawrence E. O'Rahilly S. (2004). Congenital leptin deficiency due to homozygosity for the Delta133G mutation: report of another case and evaluation of response to four years of leptin therapy. J. Clin. Endocrinol. Metab. 89, 4821–4826 10.1210/jc.2004-0376 15472169

[B66] Golub M. S. Collman G. W. Foster P. M. Kimmel C. A. Rajpert-De Meyts E. Reiter E. O. (2008). Public health implications of altered puberty timing. Pediatrics 121(Suppl. 3), S218–S230 10.1542/peds.2007-1813G 18245514

[B67] Gonzalez-Martinez D. De Mees C. Douhard Q. Szpirer C. Bakker J. (2008). Absence of gonadotropin-releasing hormone 1 and Kiss1 activation in alpha-fetoprotein knockout mice: prenatal estrogens defeminize the potential to show preovulatory luteinizing hormone surges. Endocrinology 149, 2333–2340 10.1210/en.2007-1422 18202134 PMC2329285

[B68] Goodman R. L. Lehman M. N. Smith J. T. Coolen L. M. de Oliveira C. V. Jafarzadehshirazi M. R. (2007). Kisspeptin neurons in the arcuate nucleus of the ewe express both dynorphin A and neurokinin B. Endocrinology 148, 5752–5760 10.1210/en.2007-0961 17823266

[B69] Gottsch M. L. Cunningham M. J. Smith J. T. Popa S. M. Acohido B. V. Crowley W. F. (2004). A role for kisspeptins in the regulation of gonadotropin secretion in the mouse. Endocrinology 145, 4073–4077 10.1210/en.2004-0431 15217982

[B71] Guerriero K. A. Keen K. L. Millar R. P. Terasawa E. (2012). Developmental changes in GnRH release in response to kisspeptin agonist and antagonist in female rhesus monkeys (*Macaca mulatta*): implication for the mechanism of puberty. Endocrinology 153, 825–836 10.1210/en.2011-1565 22166978 PMC3275383

[B72] Guran T. Tolhurst G. Bereket A. Rocha N. Porter K. Turan S. (2009). Hypogonadotropic hypogonadism due to a novel missense mutation in the first extracellular loop of the neurokinin B receptor. J. Clin. Endocrinol. Metab. 94, 3633–3639 10.1210/jc.2009-0551 19755480 PMC4306717

[B73] Halaas J. L. Gajiwala K. S. Maffei M. Cohen S. L. Chait B. T. Rabinowitz D. (1995). Weight-reducing effects of the plasma protein encoded by the obese gene. Science 269, 543–546 7624777 10.1126/science.7624777

[B74] He C. Kraft P. Chen C. Buring J. E. Pare G. Hankinson S. E. (2009). Genome-wide association studies identify loci associated with age at menarche and age at natural menopause. Nat. Genet. 41, 724–728 10.1038/ng.385 19448621 PMC2888798

[B75] Herbison A. E. de Tassigny X. Doran J. Colledge W. H. (2010). Distribution and postnatal development of Gpr54 gene expression in mouse brain and gonadotropin-releasing hormone neurons. Endocrinology 151, 312–321 10.1210/en.2009-0552 19966188

[B76] Herbison A. E. Porteous R. Pape J. R. Mora J. M. Hurst P. R. (2008). Gonadotropin-releasing hormone neuron requirements for puberty, ovulation, and fertility. Endocrinology 149, 597–604 10.1210/en.2007-1139 18006629 PMC6101186

[B77] Herbison A. E. Theodosis D. T. (1992). Localization of oestrogen receptors in preoptic neurons containing neurotensin but not tyrosine hydroxylase, cholecystokinin or luteinizing hormone-releasing hormone in the male and female rat. Neuroscience 50, 283–298 10.1016/0306-4522(92)90423-Y 1359459

[B78] Homma T. Sakakibara M. Yamada S. Kinoshita M. Iwata K. Tomikawa J. (2009). Significance of neonatal testicular sex steroids to defeminize anteroventral periventricular kisspeptin neurons and the GnRH/LH surge system in male rats. Biol. Reprod. 81, 1216–1225 10.1095/biolreprod.109.078311 19684332

[B79] Hrabovszky E. Ciofi P. Vida B. Horvath M. C. Keller E. Caraty A. (2010). The kisspeptin system of the human hypothalamus: sexual dimorphism and relationship with gonadotropin-releasing hormone and neurokinin B neurons. Eur. J. Neurosci. 31, 1984–1998 10.1111/j.1460-9568.2010.07239.x 20529119

[B80] Hrabovszky E. Vida B. Horvath M. C. Keller E. Caraty A. Clive C. W. (2008). Distribution of kisspeptin-like immunoreactivity in the human hypothalamus: demonstration of neuronal contacts with type-I gonadotropin-releasing hormone neurons in Proceedings of the First World Conference on Kisspeptin Signaling in the Brain (Cordoba, Spain), 73

[B81] Huang X. Harlan R. E. (1993). Absence of androgen receptors in LHRH immunoreactive neurons. Brain Res. 624, 309–311 10.1016/0006-8993(93)90094-4 8252407

[B82] Irwig M. S. Fraley G. S. Smith J. T. Acohido B. V. Popa S. M. Cunningham M. J. (2004). Kisspeptin activation of gonadotropin releasing hormone neurons and regulation of KiSS-1 mRNA in the male rat. Neuroendocrinology 80, 264–272 10.1159/000083140 15665556

[B83] Iuliano-Burns S. Hopper J. Seeman E. (2009). The age of puberty determines sexual dimorphism in bone structure: a male/female co-twin control study. J. Clin. Endocrinol. Metab. 94, 1638–1643 10.1210/jc.2008-1522 19258406

[B84] Jayasena C. N. Nijher G. M. Abbara A. Murphy K. G. Lim A. Patel D. (2010). Twice-weekly administration of kisspeptin-54 for 8 weeks stimulates release of reproductive hormones in women with hypothalamic amenorrhea. Clin. Pharmacol. Ther. 88, 840–847 10.1038/clpt.2010.204 20980998

[B85] Jayasena C. N. Nijher G. M. Chaudhri O. B. Murphy K. G. Ranger A. Lim A. (2009). Subcutaneous injection of kisspeptin-54 acutely stimulates gonadotropin secretion in women with hypothalamic amenorrhea, but chronic administration causes tachyphylaxis. J. Clin. Endocrinol. Metab. 94, 4315–4323 10.1210/jc.2009-0406 19820030

[B86] Jayasena C. N. Nijher G. M. Comninos A. N. Abbara A. Januszewki A. Vaal M. L. (2011). The effects of kisspeptin-10 on reproductive hormone release show sexual dimorphism in humans. J. Clin. Endocrinol. Metab. 96, E1963–E1972 10.1210/jc.2011-1408 21976724 PMC3232613

[B87] Kalamatianos T. Grimshaw S. E. Poorun R. Hahn J. D. Coen C. W. (2008). Fasting reduces KiSS-1 expression in the anteroventral periventricular nucleus (AVPV): effects of fasting on the expression of KiSS-1 and neuropeptide Y in the AVPV or arcuate nucleus of female rats. J. Neuroendocrinol. 20, 1089–1097 10.1111/j.1365-2826.2008.01757.x 18573184

[B88] Kaplowitz P. B. (2008). Link between body fat and the timing of puberty. Pediatrics 121(Suppl. 3), S208–S217 10.1542/peds.2007-1813F 18245513

[B89] Kaplowitz P. B. Slora E. J. Wasserman R. C. Pedlow S. E. Herman-Giddens M. E. (2001). Earlier onset of puberty in girls: relation to increased body mass index and race. Pediatrics 108, 347–353 10.1542/peds.108.2.347 11483799

[B90] Kauffman A. S. (2010). Gonadal and nongonadal regulation of sex differences in hypothalamic Kiss1 neurones. J. Neuroendocrinol. 22, 682–691 10.1111/j.1365-2826.2010.02030.x 20492362 PMC3096441

[B91] Kauffman A. S. Gottsch M. L. Roa J. Byquist A. C. Crown A. Clifton D. K. (2007a). Sexual differentiation of Kiss1 gene expression in the brain of the rat. Endocrinology 148, 1774–1783 10.1210/en.2006-1540 17204549

[B92] Kauffman A. S. Park J. H. McPhie-Lalmansingh A. A. Gottsch M. L. Bodo C. Hohmann J. G. (2007b). The kisspeptin receptor GPR54 is required for sexual differentiation of the brain and behavior. J. Neurosci. 27, 8826–8835 10.1523/JNEUROSCI.2099-07.2007 17699664 PMC6672184

[B93] Kauffman A. S. Navarro V. M. Kim J. Clifton D. K. Steiner R. A. (2009). Sex differences in the regulation of Kiss1/NKB neurons in juvenile mice: implications for the timing of puberty. Am. J. Physiol. Endocrinol. Metab. 297, E1212–E1221 10.1152/ajpendo.00461.2009 19755669 PMC2781353

[B94] Keen K. L. Burich A. J. Mitsushima D. Kasuya E. Terasawa E. (1999). Effects of pulsatile infusion of the GABA(A) receptor blocker bicuculline on the onset of puberty in female rhesus monkeys. Endocrinology 140, 5257–5266 10.1210/en.140.11.5257 10537156

[B95] Kennedy G. C. Mitra J. (1963). Body weight and food intake as initiating factors for puberty in the rat. J. Physiol. 166, 408–418 14031944 10.1113/jphysiol.1963.sp007112 PMC1359337

[B96] Kiess W. Blum W. F. Aubert M. L. (1998). Leptin, puberty and reproductive function: lessons from animal studies and observations in humans. Eur. J. Endocrinol. 138, 26–29 10.1530/eje.0.1380026 9461310

[B97] Kim G. L. Dhillon S. S. Belsham D. D. (2010). Kisspeptin directly regulates neuropeptide Y synthesis and secretion via the ERK1/2 and p38 mitogen-activated protein kinase signaling pathways in NPY-secreting hypothalamic neurons. Endocrinology 151, 5038–5047 10.1210/en.2010-0521 20685868

[B98] Klein K. O. (1999). Precocious puberty: who has it? Who should be treated? J. Clin. Endocrinol. Metab. 84, 411–414 10.1210/jc.84.2.411 10022393

[B99] Krey L. C. Butler W. R. Knobil E. (1975). Surgical disconnection of medial basal hypothalamus and pituitary-function in rhesus-monkey.1. Gonadotropin-Secretion. Endocrinology 96, 1073–1087 10.1210/endo-96-5-1073 804398

[B100] Kurian J. R. Keen K. L. Guerriero K. A. Terasawa E. (2012). Tonic control of kisspeptin release in prepubertal monkeys: implications to the mechanism of puberty onset. Endocrinology 153, 3331–3336 10.1210/en.2012-1221 22585828 PMC3380308

[B101] Lanfranco F. Gromoll J. von Eckardstein S. Herding E. M. Nieschlag E. Simoni M. (2005). Role of sequence variations of the GnRH receptor and G protein-coupled receptor 54 gene in male idiopathic hypogonadotropic hypogonadism. Eur. J. Endocrinol. 153, 845–852 10.1530/eje.1.02031 16322390

[B102] Lapatto R. Pallais J. C. Zhang D. Chan Y. M. Mahan A. Cerrato F. (2007). Kiss1-/- mice exhibit more variable hypogonadism than Gpr54-/- mice. Endocrinology 148, 4927–4936 10.1210/en.2007-0078 17595229

[B103] Lavoie H. B. Taylor A. E. Sharpless J. L. Anderson E. J. Strauss C. C. Hall J. E. (1999). Effects of short-term hormone replacement on serum leptin levels in postmenopausal women. Clin. Endocrinol. (Oxf.) 51, 415–422 10.1046/j.1365-2265.1999.00796.x 10583307

[B104] Lee J. M. Appugliese D. Kaciroti N. Corwyn R. F. Bradley R. H. Lumeng J. C. (2007). Weight status in young girls and the onset of puberty. Pediatrics 119, e624–e630 10.1542/peds.2006-2188 17332182

[B105] Lee K. Porteous R. Campbell R. E. Luscher B. Herbison A. E. (2010). Knockdown of GABA(A) receptor signaling in GnRH neurons has minimal effects upon fertility. Endocrinology 151, 4428–4436 10.1210/en.2010-0314 20573723 PMC5398471

[B106] Lents C. A. Heidorn N. L. Barb C. R. Ford J. J. (2008). Central and peripheral administration of kisspeptin activates gonadotropin but not somatotropin secretion in prepubertal gilts. Reproduction 135, 879–887 10.1530/REP-07-0502 18339687

[B107] Licinio J. Caglayan S. Ozata M. Yildiz B. O. de Miranda P. B. O'Kirwan F. (2004). Phenotypic effects of leptin replacement on morbid obesity, diabetes mellitus, hypogonadism, and behavior in leptin-deficient adults. Proc. Natl. Acad. Sci. U.S.A. 101, 4531–4536 10.1073/pnas.0308767101 15070752 PMC384781

[B108] Licinio J. Negrao A. B. Mantzoros C. Kaklamani V. Wong M. L. Bongiorno P. B. (1998). Synchronicity of frequently sampled, 24-h concentrations of circulating leptin, luteinizing hormone, and estradiol in healthy women. Proc. Natl. Acad. Sci. U.S.A. 95, 2541–2546 9482922 10.1073/pnas.95.5.2541 PMC19406

[B109] Lin L. Ercan O. Raza J. Burren C. P. Creighton S. M. Auchus R. J. (2007). Variable phenotypes associated with aromatase (CYP19) insufficiency in humans. J. Clin. Endocrinol. Metab. 92, 982–990 10.1210/jc.2006-1181 17164303 PMC1955738

[B110] Luan X. Yu H. Wei X. Zhou Y. Wang W. Li P. (2002). GPR54 polymorphisms in Chinese girls with central precocious puberty. Neuroendocrinology 86, 77–83 10.1159/000107511 17700012

[B111] Luan X. Zhou Y. Wang W. Yu H. Li P. Gan X. (2007). Association study of the polymorphisms in the KISS1 gene with central precocious puberty in Chinese girls. Eur. J. Endocrinol. 157, 113–118 10.1530/EJE-07-0061 17609410

[B112] Luque R. M. Kineman R. D. Tena-Sempere M. (2007). Regulation of hypothalamic expression of KiSS-1 and GPR54 genes by metabolic factors: analyses using mouse models and a cell line. Endocrinology 148, 4601–4611 10.1210/en.2007-0500 17595226

[B113] Mann D. R. Akinbami M. A. Gould K. G. Paul K. Wallen K. (1998). Sexual maturation in male rhesus monkeys: importance of neonatal testosterone exposure and social rank. J. Endocrinol. 156, 493–501 10.1677/joe.0.1560493 9582506

[B114] Matkovic V. Ilich J. Z. Badenhop N. E. Skugor M. Clairmont A. Klisovic D. (1997). Gain in body fat is inversely related to the nocturnal rise in serum leptin level in young females. J. Clin. Endocrinol. Metab. 82, 1368–1372 10.1210/jc.82.5.1368 9141517

[B115] Matsui H. Takatsu Y. Kumano S. Matsumoto H. Ohtaki T. (2004). Peripheral administration of metastin induces marked gonadotropin release and ovulation in the rat. Biochem. Biophys. Res. Commun. 320, 383–388 10.1016/j.bbrc.2004.05.185 15219839

[B116] Mayer C. Acosta-Martinez M. Dubois S. L. Wolfe A. Radovick S. Boehm U. (2010). Timing and completion of puberty in female mice depend on estrogen receptor alpha-signaling in kisspeptin neurons. Proc. Natl. Acad. Sci. U.S.A. 107, 22693–22698 10.1073/pnas.1012406108 21149719 PMC3012491

[B117] McCarthy M. M. (2008). Estradiol, and the developing brain. Physiol. Rev. 88, 91–124 10.1152/physrev.00010.2007 18195084 PMC2754262

[B118] McCarthy M. M. Wright C. L. Schwarz J. M. (2009). New tricks by an old dogma: mechanisms of the Organizational/Activational Hypothesis of steroid-mediated sexual differentiation of brain and behavior. Horm. Behav. 55, 655–665 10.1016/j.yhbeh.2009.02.012 19682425 PMC2742630

[B119] Messager S. Chatzidaki E. E. Ma D. Hendrick A. G. Zahn D. Dixon J. (2005). Kisspeptin directly stimulates gonadotropin-releasing hormone release via G protein-coupled receptor 54. Proc. Natl. Acad. Sci. U.S.A. 102, 1761–1766 10.1073/pnas.0409330102 15665093 PMC545088

[B120] Michael R. P. Bonsall R. W. Rees H. D. (1987). Sites at which testosterone may act as an estrogen in the brain of the male primate. Neuroendocrinology 46, 511–521 3122067 10.1159/000124874

[B121] Mikkelsen J. D. Bentsen A. H. Ansel L. Simonneaux V. Juul A. (2009). Comparison of the effects of peripherally administered kisspeptins. Regul. Pept. 152, 95–100 10.1016/j.regpep.2008.10.001 18940206

[B122] Millar R. P. Roseweir A. K. Tello J. A. Anderson R. A. George J. T. Morgan K. (2010). Kisspeptin antagonists: unraveling the role of kisspeptin in reproductive physiology. Brain Res. 1364, 81–89 10.1016/j.brainres.2010.09.044 20858467

[B123] Mohamed J. S. Benninghoff A. D. Holt G. J. Khan I. A. (2007). Developmental expression of the G protein-coupled receptor 54 and three GnRH mRNAs in the teleost fish cobia. J. Mol. Endocrinol. 38, 235–244 10.1677/jme.1.02182 17293443

[B124] Montague C. T. Farooqi I. S. Whitehead J. P. Soos M. A. Rau H. Wareham N. J. (1977). Congenital leptin deficiency is associated with severe early-onset obesity in humans. Nature 387, 903–908 10.1038/43185 9202122

[B125] Mounzih K. Lu R. Chehab F. F. (1997). Leptin treatment rescues the sterility of genetically obese ob/ob males. Endocrinology 138, 1190–1193 10.1210/en.138.3.1190 9048626

[B126] Nagatani S. Guthikonda P. Thompson R. C. Tsukamura H. Maeda K. I. Foster D. L. (1998). Evidence for GnRH regulation by leptin: leptin administration prevents reduced pulsatile LH secretion during fasting. Neuroendocrinology 67, 370–376 9662716 10.1159/000054335

[B127] Navarro V. M. Castellano J. M. Fernandez-Fernandez R. Barreiro M. L. Roa J. Sanchez-Criado J. E. (2004a). Developmental and hormonally regulated messenger ribonucleic acid expression of KiSS-1 and its putative receptor, GPR54, in rat hypothalamus and potent luteinizing hormone-releasing activity of KiSS-1 peptide. Endocrinology 145, 4565–4574 10.1210/en.2004-0413 15242985

[B128] Navarro V. M. Fernandez-Fernandez R. Castellano J. M. Roa J. Mayen A. Barreiro M. L. (2004b). Advanced vaginal opening and precocious activation of the reproductive axis by KiSS-1 peptide, the endogenous ligand of GPR54. J. Physiol. 561, 379–386 10.1113/jphysiol.2004.072298 15486019 PMC1665361

[B129] Navarro V. M. Castellano J. M. Fernandez-Fernandez R. Tovar S. Roa J. Mayen A. (2005). Effects of KiSS-1 peptide, the natural ligand of GPR54, on follicle-stimulating hormone secretion in the rat. Endocrinology 146, 1689–1697 10.1210/en.2004-1353 15637288

[B130] Navarro V. M. Gottsch M. L. Chavkin C. Okamura H. Clifton D. K. Steiner R. A. (2009). Regulation of gonadotropin-releasing hormone secretion by kisspeptin/dynorphin/neurokinin B neurons in the arcuate nucleus of the mouse. J. Neurosci. 29, 11859–11866 10.1523/JNEUROSCI.1569-09.2009 19776272 PMC2793332

[B131] Navarro V. M. Gottsch M. L. Wu M. Garca-Galiano D. Hobbs S. J. Bosch M. A. (2011). Regulation of NKB pathways and their roles in the control of Kiss1 neurons in the arcuate nucleus of the male mouse. Endocrinology 152, 4265–4275 10.1210/en.2011-1143 21914775 PMC3198996

[B132] Nimri R. Lebenthal Y. Lazar L. Chevrier L. Phillip M. Bar M. (2011). A novel loss-of-function mutation in GPR54/KISS1R leads to hypogonadotropic hypogonadism in a highly consanguineous family. J. Clin. Endocrinol. Metab. 96, E536–E545 10.1210/jc.2010-1676 21193544

[B133] Nottelmann E. D. Susman E. J. Dorn L. D. Inoff-Germain G. Loriaux D. L. Cutler G. B. Jr. (1987). Developmental processes in early adolescence. Relations among chronologic age, pubertal stage, height, weight, and serum levels of gonadotropins, sex steroids, and adrenal androgens. J. Adolesc. Health Care 8, 246–260 3583875 10.1016/0197-0070(87)90428-1

[B134] Ogden C. L. Carroll M. D. Kit B. K. Flegal K. M. (2012). Prevalence of obesity and trends in body mass index among US children and adolescents, 1999-(2010). JAMA 307, 483–490 10.1001/jama.2012.40 22253364 PMC6362452

[B135] Ong K. K. Elks C. E. Li S. Zhao J. H. Luan J. Andersen L. B. (2009). Genetic variation in LIN28B is associated with the timing of puberty. Nat. Genet. 41, 729–733 10.1038/ng.382 19448623 PMC3000552

[B136] Ozata M. Ozdemir I. C. Licinio J. (1999). Human leptin deficiency caused by a missense mutation: multiple endocrine defects, decreased sympathetic tone, and immune system dysfunction indicate new targets for leptin action, greater central than peripheral resistance to the effects of leptin, and spontaneous correction of leptin-mediated defects. J. Clin. Endocrinol. Metab. 84, 3686–3695 10.1210/jc.84.10.3686 10523015

[B137] Pallais J. C. Bo-Abbas Y. Pitteloud N. Crowley W. F. Jr. Seminara S. B. (2006). Neuroendocrine, gonadal, placental, and obstetric phenotypes in patients with IHH and mutations in the G-protein coupled receptor, GPR54. Mol. Cell. Endocrinol. 254–255, 70–77 10.1016/j.mce.2006.04.019 16757106

[B138] Palmert M. R. Boepple P. A. (2001). Variation in the timing of puberty: clinical spectrum and genetic investigation. J. Clin. Endocrinol. Metab. 86, 2364–2368 10.1210/jc.86.6.2364 11397824

[B139] Palmert M. R. Hirschhorn J. N. (2003). Genetic approaches to stature, pubertal timing, and other complex traits. Mol. Genet. Metab. 80, 1–10 10.1016/S1096-7192(03)00107-0 14567953

[B140] Parent A. S. Teilmann G. Juul A. Skakkebaek N. E. Toppari J. Bourguignon J. P. (2003). The timing of normal puberty and the age limits of sexual precocity: variations around the world, secular trends, and changes after migration. Endocr. Rev. 24, 668–693 10.1210/er.2002-0019 14570750

[B141] Parhar I. S. Ogawa S. Sakuma Y. (2004). Laser-captured single digoxigenin-labeled neurons of gonadotropin-releasing hormone types reveal a novel G protein-coupled receptor (Gpr54) during maturation in cichlid fish. Endocrinology 145, 3613–3618 10.1210/en.2004-0395 15155576

[B142] Perry J. R. Stolk L. Franceschini N. Lunetta K. L. Zhai G. McArdle P. F. (2009). Meta-analysis of genome-wide association data identifies two loci influencing age at menarche. Nat. Genet. 41, 648–650 10.1038/ng.386 19448620 PMC2942986

[B143] Pheng V. Uenoyama Y. Homma T. Inamoto Y. Takase K. Yoshizawa-Kumagaye K. (2009). Potencies of centrally- or peripherally-injected full-length kisspeptin or its C-terminal decapeptide on LH release in intact male rats. J. Reprod. Dev. 55, 378–382 19384054 10.1262/jrd.20240

[B144] Phoenix C. H. Goy R. W. Gerall A. A. Young W. C. (1959). Organizing action of prenatally administered testosterone propionate on the tissues mediating mating behavior in the female guinea pig. Endocrinology 65, 369–382 10.1016/j.yhbeh.2009.03.015 14432658

[B145] Pita J. Barrios V. Gavela-Perez T. Martos-Moreno G. A. Munoz-Calvo M. T. Pozo J. (2011a). Circulating kisspeptin levels exhibit sexual dimorphism in adults, are increased in obese prepubertal girls and do not suffer modifications in girls with idiopathic central precocious puberty. Peptides 32, 1781–1786 10.1016/j.peptides.2011.07.016 21827808

[B146] Pita J. Rado-Peralta S. Gavela-Perez T. Aragon I. Barrios V. Rovira A. (2011b). Plasma kisspeptin levels are elevated in cord blood and present sexual dimorphism in the adult population: relation with leptin, gonadotropins and anthropometrical data. Peptides 32, 983–988 10.1016/j.peptides.2011.01.030 21295095

[B147] Plant T. M. Gay V. L. Marshall G. R. Arslan M. (1989). Puberty in monkeys is triggered by chemical stimulation of the hypothalamus. Proc. Natl. Acad. Sci. U.S.A. 86, 2506–2510 2648405 10.1073/pnas.86.7.2506 PMC286942

[B148] Plant T. M. Krey L. C. Moossy J. McCormack J. T. Hess D. L. Knobil E. (1978). The arcuate nucleus and the control of gonadotropin and prolactin secretion in the female rhesus monkey (*Macaca mulatta*). Endocrinology 102, 52–62 10.1210/endo-102-1-52 105866

[B149] Prete G. Couto-Silva A. C. Trivin C. Brauner R. (2008). Idiopathic central precocious puberty in girls: presentation factors. BMC Pediatr. 8:27 10.1186/1471-2431-8-27 18601733 PMC2459158

[B150] Quennell J. H. Howell C. S. Roa J. Augustine R. A. Grattan D. R. Anderson G. M. (2011). Leptin deficiency and diet-induced obesity reduce hypothalamic kisspeptin expression in mice. Endocrinology 152, 1541–1550 10.1210/en.2010-1100 21325051 PMC3206710

[B151] Ramaswamy S. Seminara S. B. Ali B. Ciofi P. Amin N. A. Plant T. M. (2010). Neurokinin B stimulates GnRH release in the male monkey (*Macaca mulatta*) and is colocalized with kisspeptin in the arcuate nucleus. Endocrinology 151, 4494–4503 10.1210/en.2010-0223 20573725 PMC2940495

[B152] Ramaswamy S. Seminara S. B. Pohl C. R. DiPietro M. J. Crowley W. F. Jr. Plant T. M. (2007). Effect of continuous intravenous administration of human metastin 45–54 on the neuroendocrine activity of the hypothalamic-pituitary-testicular axis in the adult male rhesus monkey (*Macaca mulatta*). Endocrinology 148, 3364–3370 10.1210/en.2007-0207 17412800

[B153] Resko J. A. Goy R. W. Robinson J. A. Norman R. L. (1982). The pubescent rhesus monkey: some characteristics of the menstrual cycle. Biol. Reprod. 27, 354–361 10.1095/biolreprod27.2.354 7126735

[B154] Rhie Y. J. Lee K. H. Eun S. H. Choi B. M. Chae H. W. Kwon A. R. (2011). Serum kisspeptin levels in Korean girls with central precocious puberty. J. Korean Med. Sci. 26, 927–931 10.3346/jkms.2011.26.7.927 21738347 PMC3124724

[B155] Roa J. Vigo E. Garcia-Galiano D. Castellano J. M. Navarro V. M. Pineda R. (2008). Desensitization of gonadotropin responses to kisspeptin in the female rat: analyses of LH and FSH secretion at different developmental and metabolic states. J. Am. Physiol. Endocrinol. Metab. 294, E1088–E1096 10.1152/ajpendo.90240.2008 18413669

[B156] Rosenfield R. L. Lipton R. B. Drum M. L. (2009). Thelarche, pubarche, and menarche attainment in children with normal and elevated body mass index. Pediatrics 123, 84–88 10.1542/peds.2008-0146 19117864

[B157] Levina S. E. (1972). Times of appearance of LH and FSH activities in human fetal circulation. Gen. Comp. Endocrinol. 19, 242–246 10.1016/0016-6480(72)90102-5 4635457

[B158] Schwanzel-Fukuda M. Robinson J. A. Silverman A. J. (1981). The fetal development of the luteinizing hormone-releasing hormone (LHRH) neuronal systems of the guinea pig brain. Brain Res. Bull. 7, 293–315 7023618 10.1016/0361-9230(81)90021-6

[B159] Seminara S. B. Dipietro M. J. Ramaswamy S. Crowley W. F. Jr. Plant T. M. (2006). Continuous human metastin 45-54 infusion desensitizes G protein-coupled receptor 54-induced gonadotropin-releasing hormone release monitored indirectly in the juvenile male Rhesus monkey (*Macaca mulatta*): a finding with therapeutic implications. Endocrinology 147, 2122–2126 10.1210/en.2005-1550 16469799

[B160] Seminara S. B. Hayes F. J. Crowley W. F. Jr. (1998). Gonadotropin-releasing hormone deficiency in the human (idiopathic hypogonadotropic hypogonadism and Kallmann's syndrome): pathophysiological and genetic considerations. Endocr. Rev. 19, 521–539 10.1210/er.19.5.521 9793755

[B161] Seminara S. B. Messager S. Chatzidaki E. E. Thresher R. R. Acierno J. S. Jr. Shagoury J. K. (2003). The GPR54 gene as a regulator of puberty. N. Engl. J. Med. 349, 1614–1627 10.1056/NEJMoa035322 14573733

[B162] Semple R. K. Achermann J. C. Ellery J. Farooqi I. S. Karet F. E. Stanhope R. G. (2005). Two novel missense mutations in g protein-coupled receptor 54 in a patient with hypogonadotropic hypogonadism. J. Clin. Endocrinol. Metab. 90, 1849–1855 10.1210/jc.2004-1418 15598687

[B163] Shahab M. Mastronardi C. Seminara S. B. Crowley W. F. Ojeda S. R. Plant T. M. (2005). Increased hypothalamic GPR54 signaling: a potential mechanism for initiation of puberty in primates. Proc. Natl. Acad. Sci. U.S.A. 102, 2129–2134 10.1073/pnas.0409822102 15684075 PMC548549

[B164] Silveira L. G. Noel S. D. Silveira-Neto A. P. Abreu A. P. Brito V. N. Santos M. G. (2010). Mutations of the KISS1 gene in disorders of puberty. J. Clin. Endocrinol. Metab. 95, 2276–2280 10.1210/jc.2009-2421 20237166 PMC2869552

[B165] Simerly R. B. (2002). Wired for reproduction: organization and development of sexually dimorphic circuits in the mammalian forebrain. Annu. Rev. Neurosci. 25, 507–536 10.1146/annurev.neuro.25.112701.142745 12052919

[B166] Smith J. T. Acohido B. V. Clifton D. K. Steiner R. A. (2006a). KiSS-1 neurones are direct targets for leptin in the ob/ob mouse. J. Neuroendocrinol. 18, 298–303 10.1111/j.1365-2826.2006.01417.x 16503925

[B167] Smith J. T. Clifton D. K. Steiner R. A. (2006b). Regulation of the neuroendocrine reproductive axis by kisspeptin-GPR54 signaling. Reproduction 131, 623–630 10.1530/rep.1.00368 16595713

[B168] Smith J. T. Clay C. M. Caraty A. Clarke I. J. (2007). KiSS-1 messenger ribonucleic acid expression in the hypothalamus of the ewe is regulated by sex steroids and season. Endocrinology 148, 1150–1157 10.1210/en.2006-1435 17185374

[B169] Smith J. T. Cunningham M. J. Rissman E. F. Clifton D. K. Steiner R. A. (2005a). Regulation of Kiss1 gene expression in the brain of the female mouse. Endocrinology 146, 3686–3692 10.1210/en.2005-0488 15919741

[B170] Smith J. T. Dungan H. M. Stoll E. A. Gottsch M. L. Braun R. E. Eacker S. M. (2005b). Differential regulation of KiSS-1 mRNA expression by sex steroids in the brain of the male mouse. Endocrinology 146, 2976–2984 10.1210/en.2005-0323 15831567

[B171] Strobel A. Issad T. Camoin L. Ozata M. Strosberg A. D. (1998). A leptin missense mutation associated with hypogonadism and morbid obesity. Nat. Genet. 18, 213–215 10.1038/ng0398-213 9500540

[B172] Swerdloff R. S. Batt R. A. Bray G. A. (1976). Reproductive hormonal function in the genetically obese (ob/ob) mouse. Endocrinology 98, 1359–1364 10.1210/endo-98-6-1359 1278106

[B173] Sykiotis G. P. Hoang X. H. Avbelj M. Hayes F. J. Thambundit A. Dwyer A. (2010). Congenital idiopathic hypogonadotropic hypogonadism: evidence of defects in the hypothalamus, pituitary, and testes. J. Clin. Endocrinol. Metab. 95, 3019–3027 10.1210/jc.2009-2582 20382682 PMC2902061

[B174] Takumi K. Iijima N. Ozawa H. (2011). Developmental changes in the expression of kisspeptin mRNA in rat hypothalamus. J. Mol. Neurosci. 43, 138–145 10.1007/s12031-010-9430-1 20665248

[B175] Teilmann G. Pedersen C. B. Jensen T. K. Skakkebaek N. E. Juul A. (2005). Prevalence and incidence of precocious pubertal development in Denmark: an epidemiologic study based on national registries. Pediatrics 116, 1323–1328 10.1542/peds.2005-0012 16322154

[B176] Teles M. G. Bianco S. D. Brito V. N. Trarbach E. B. Kuohung W. Xu S. (2008). A GPR54-activating mutation in a patient with central precocious puberty. N. Engl. J. Med. 358, 709–715 10.1056/NEJMoa073443 18272894 PMC2859966

[B177] Teles M. G. Silveira L. F. Tusset C. Latronico A. C. (2011). New genetic factors implicated in human GnRH-dependent precocious puberty: the role of kisspeptin system. Mol. Cell. Endocrinol. 346, 84–90 10.1016/j.mce.2011.05.019 21664234

[B178] Teles M. G. Trarbach E. B. Noel S. D. Guerra-Junior G. Jorge A. Beneduzzi D. (2010). A novel homozygous splice acceptor site mutation of KISS1R in two siblings with normosmic isolated hypogonadotropic hypogonadism. Eur. J. Endocrinol. 163, 29–34 10.1530/EJE-10-0012 20371656

[B179] Tenenbaum-Rakover Y. Commenges-Ducos M. Iovane A. Aumas C. Admoni O. de Roux N. (2007). Neuroendocrine phenotype analysis in five patients with isolated hypogonadotropic hypogonadism due to a L102P inactivating mutation of GPR54. J. Clin. Endocrinol. Metab. 92, 1137–1144 10.1210/jc.2006-2147 17164310

[B180] Terasawa E. Fernandez D. L. (2001). Neurobiological mechanisms of the onset of puberty in primates. Endocr. Rev. 22, 111–151 10.1210/er.22.1.111 11159818

[B181] Terasawa E. Kurian J. R. Guerriero K. A. Kenealy B. P. Hutz E. D. Keen K. L. (2010). Recent discoveries on the control of gonadotrophin-releasing hormone neurones in nonhuman primates. J. Neuroendocrinol. 22, 630–638 10.1111/j.1365-2826.2010.02027.x 20456608 PMC2908205

[B182] Topaloglu A. K. Lu Z. L. Farooqi I. S. Mungan N. O. Yuksel B. O'Rahilly S. (2006). Molecular genetic analysis of normosmic hypogonadotropic hypogonadism in a Turkish population: identification and detailed functional characterization of a novel mutation in the gonadotropin-releasing hormone receptor gene. Neuroendocrinology 84, 301–308 10.1159/000098147 17179725

[B183] Topaloglu A. K. Reimann F. Guclu M. Yalin A. S. Kotan L. D. Porter K. M. (2009). TAC3 and TACR3 mutations in familial hypogonadotropic hypogonadism reveal a key role for Neurokinin B in the central control of reproduction. Nat. Genet. 41, 354–358 10.1038/ng.306 19079066 PMC4312696

[B184] Topaloglu A. K. Tello J. A. Kotan L. D. Ozbek M. N. Yilmaz M. B. Erdogan S. (2012). Inactivating KISS1 mutation and hypogonadotropic hypogonadism. N. Engl. J. Med. 366, 629–635 10.1056/NEJMoa1111184 22335740

[B185] Tusset C. (2010). Mutaitonal analysis of TAC3 and TACR3 genes in chidlren with idiopathic cetral precocious puberty, in The 92th Annual Meeting of the Endocrine Society (San Diego, CA), S773

[B186] Wakabayashi Y. Nakada T. Murata K. Ohkura S. Mogi K. Navarro V. M. (2010). Neurokinin B and dynorphin A in kisspeptin neurons of the arcuate nucleus participate in generation of periodic oscillation of neural activity driving pulsatile gonadotropin-releasing hormone secretion in the goat. J. Neurosci. 30, 3124–3132 10.1523/JNEUROSCI.5848-09.2010 20181609 PMC6633939

[B187] Wallen K. (2005). Hormonal influences on sexually differentiated behavior in nonhuman primates. Front. Neuroendocrinol. 26:1 10.1016/j.yfrne.2005.02.001 15862182

[B188] Walvoord E. C. (2010). The timing of puberty: is it changing? Does it matter? J. Adolesc. Health 47, 433–439 10.1016/j.jadohealth.2010.05.018 20970077

[B189] Wang Y. (2002). Is obesity associated with early sexual maturation? A comparison of the association in American boys versus girls. Pediatrics 110, 903–910 10.1542/peds.110.5.903 12415028

[B190] Wattigney W. A. Srinivasan S. R. Chen W. Greenlund K. J. Berenson G. S. (1999). Secular trend of earlier onset of menarche with increasing obesity in black and white girls: the Bogalusa Heart Study. Ethn. Dis. 9, 181–189 10421080

[B191] Welt C. K. Chan J. L. Bullen J. Murphy R. Smith P. DePaoli A. M. (2004). Recombinant human leptin in women with hypothalamic amenorrhea. N. Engl. J. Med. 351, 987–997 10.1056/NEJMoa040388 15342807

[B192] Wilen R. Naftolin F. (1976). Age, weight and weight gain in the individual pubertal female rhesus monkey (*Macaca mulatta*). Biol. Reprod. 15, 356–360 10.1095/biolreprod15.3.356 822891

[B193] Witham E. A. Meadows J. D. Shojaei S. Kauffman A. S. Mellon P. L. (2012). Prenatal exposure to low levels of androgen accelerates female puberty onset and reproductive senescence in mice. Endocrinology 153, 4522–4532 10.1210/en.2012-1283 22778229 PMC3423623

[B194] Wray S. Gainer H. (1987). Effect of neonatal gonadectomy on the postnatal development of LHRH cell subtypes in male and female rats. Neuroendocrinology 45, 413–419 3295575 10.1159/000124767

[B195] Wu F. C. Butler G. E. Kelnar C. J. Stirling H. F. Huhtaniemi I. (1991). Patterns of pulsatile luteinizing hormone and follicle-stimulating hormone secretion in prepubertal (midchildhood) boys and girls and patients with idiopathic hypogonadotropic hypogonadism (Kallmann's syndrome): a study using an ultrasensitive time-resolved immunofluorometric assay. J. Clin. Endocrinol. Metab. 72, 1229–1237 10.1210/jcem-72-6-1229 1902843

[B196] Young J. Bouligand J. Francou B. Raffin-Sanson M. L. Gaillez S. Jeanpierre M. (2010). TAC3 and TACR3 defects cause hypothalamic congenital hypogonadotropic hypogonadism in humans. J. Clin. Endocrinol. Metab. 95, 2287–2295 10.1210/jc.2009-2600 20194706

[B197] Zirilli L. Rochira V. Diazzi C. Caffagni G. Carani C. (2008). Human models of aromatase deficiency. J. Steroid Biochem. Mol. Biol. 109, 212–218 10.1016/j.jsbmb.2008.03.026 18448329

